# Characterization of genetic rearrangements in esophageal squamous carcinoma cell lines by a combination of M-FISH and array-CGH: further confirmation of some split genomic regions in primary tumors

**DOI:** 10.1186/1471-2407-12-367

**Published:** 2012-08-24

**Authors:** Jia-Jie Hao, Zhi-Zhou Shi, Zhi-Xin Zhao, Yu Zhang, Ting Gong, Chun-Xiang Li, Ting Zhan, Yan Cai, Jin-Tang Dong, Song-Bin Fu, Qi-Min Zhan, Ming-Rong Wang

**Affiliations:** 1 State Key Laboratory of Molecular Oncology, Cancer Institute (Hospital), Peking Union Medical College and Chinese Academy of Medical Science, 17 Panjiayuan Nanli, Chaoyang District, Beijing, 100021, China; 2 Pathology department, Tongliao Hospital, 16 Keerqin Road, Tongliao, 028000, China; 3 Laboratory of Medical Genetics, Harbin Medical University, Harbin, 150081, China; 4 Winship Cancer Institute, Emory University School of Medicine, 1365-C Clifton Road, Atlanta, GA, 30322, USA

## Abstract

**Background:**

Chromosomal and genomic aberrations are common features of human cancers. However, chromosomal numerical and structural aberrations, breakpoints and disrupted genes have yet to be identified in esophageal squamous cell carcinoma (ESCC).

**Methods:**

Using multiplex-fluorescence in situ hybridization (M-FISH) and oligo array-based comparative hybridization (array-CGH), we identified aberrations and breakpoints in six ESCC cell lines. Furthermore, we detected recurrent breakpoints in primary tumors by dual-color FISH.

**Results:**

M-FISH and array-CGH results revealed complex numerical and structural aberrations. Frequent gains occurred at 3q26.33-qter, 5p14.1-p11, 7pter-p12.3, 8q24.13-q24.21, 9q31.1-qter, 11p13-p11, 11q11-q13.4, 17q23.3-qter, 18pter-p11, 19 and 20q13.32-qter. Losses were frequent at 18q21.1-qter. Breakpoints that clustered within 1 or 2 Mb were identified, including 9p21.3, 11q13.3-q13.4, 15q25.3 and 3q28. By dual-color FISH, we observed that several recurrent breakpoint regions in cell lines were also present in ESCC tumors. In particular, breakpoints clustered at 11q13.3-q13.4 were identified in 43.3% (58/134) of ESCC tumors. Both 11q13.3-q13.4 splitting and amplification were significantly correlated with lymph node metastasis (LNM) (*P* = 0.004 and 0.022) and advanced stages (*P* = 0.004 and 0.039). Multivariate logistic regression analysis revealed that only 11q13.3-q13.4 splitting was an independent predictor for LNM (*P* = 0.026).

**Conclusions:**

The combination of M-FISH and array-CGH helps produce more accurate karyotypes. Our data provide significant, detailed information for appropriate uses of these ESCC cell lines for cytogenetic and molecular biological studies. The aberrations and breakpoints detected in both the cell lines and primary tumors will contribute to identify affected genes involved in the development and progression of ESCC.

## Background

Chromosomal and genomic rearrangements are significant features of malignant human tumors. Rearrangements are often associated with structural aberrations, such as translocations, insertions and inversions. They could also result in the copy number alterations (CNAs) [1,2]. Characterizing rearrangements and genes affected by the aberrations and breakpoints might help us to understand tumor development and progression better.

The products and implications of chromosomal rearrangements (e.g., fusion genes, truncated genes, and gene dysregulation by ectopic promoters) have been described in leukemia, lymphoma, sarcomas, and epithelial cancers [3,4]. It was initially difficult to detect chromosomal rearrangements and affected genes in the epithelial cancers, mainly due to the technical difficulty of preparing metaphase spreads from primary epithelial tumors and the karyotypic complexity. Until recently, multiple gene rearrangements and even genomic landscapes which reflect the structural aberrations throughout the genomes have been identified in multiple types of epithelial cancers, including prostate cancer [5,6], breast cancer [7,8], lung cancer [9,10], colorectal cancer [11], gastric cancer [12], head and neck cancer [13], hepatocellular carcinoma [14] and so on.

Recently, it has been reported that recurrent rearrangements could affect genes at the boundaries of CNAs [2,15], thus recurrent breakpoints might be important for screening and identifying frequent unbalanced rearrangements and the involved genes. Multiplex-fluorescence in situ hybridization (M-FISH) [16] and spectral karyotyping (SKY) [17] were designed to replace traditional G-banding in chromosomal analyses of tumor cells, but the resolution of these techniques is not sufficient to detect small rearrangements. Array-based comparative genomic hybridization (array-CGH) was developed to analyze the CNAs, including genomic gains, losses, amplifications and deletions [18,19]. It was recently demonstrated that array-CGH could be used to identify unbalanced breakpoints of the rearrangements in many types of cancer cells at a potentially higher resolution [20-24]. Array-CGH has also been used, in combination with cytogenetic information, to determine the breakpoints in reciprocal translocations [25].

Esophageal cancer (EC) is a common malignant epithelial cancer worldwide, causing more than 40,000 deaths each year [26]. The most prevalent type of EC is esophageal squamous cell carcinoma (ESCC), and China is among the highest risk areas [26,27]. Recently, our group reported the karyotype of ESCC cell line KYSE180 [28] and KYSE450 [29] by 12-color M-FISH, as well as the karyotype of KYSE410-4 by 6-color M-FISH [30]. CGH [31-34], SKY and CGH [35], and array-CGH [36-38] experiments from other groups have also been performed on ESCC cell lines and primary tumors. These studies have revealed numerical and structural chromosomal aberrations. However, genomic rearrangements, breakpoints and genes that are involved in ESCC remain to be decoded and clarified.

Our study intended to identify candidate recurrent breakpoints which might affect genes at or near the boundaries. In this study, we describe CNAs and unbalanced genetic rearrangements in six ESCC cell lines through a combination of M-FISH and 44K array-CGH techniques. We found recurrent breakpoint regions in the cell lines and breakage of several regions present in primary ESCC tumors, which may contribute to disruption of critical genes.

## Methods

### Cell lines and sample collection

ESCC cell lines KYSE30, KYSE150, KYSE180, KYSE450, KYSE510 and YES2 were kindly provided by Yutaka Shimada (Kyoto University, Japan). KYSE150 and KYSE510 were established from female patients, and KYSE30, KYSE180, KYSE450 and YES2 were from male patients. Each cell line was cultured in RPMI-1640 (Invitrogen, USA) supplemented with 10% fetal calf serum (FCS). ESCC tissue samples were procured from Chinese Academy of Medical Sciences Cancer Hospital. All the samples used in this study were residual specimens collected after diagnosis sampling. And all patients received no treatment before surgery, and signed separate informed consent forms for the sampling and molecular analyses. This study has been approved by the Ethics Committee/IRB of Cancer Institute (Hospital), PUMC/CAMS.

### Metaphase chromosomes and interphase cell nuclei preparations

Metaphase chromosomes from ESCC cell lines and normal peripheral blood lymphocytes were harvested after incubation with 0.04 μg/ml Colcemid (Invitrogen) at 37°C for 1-2 hours, followed by treatment with a hypotonic solution (0.075 mol/L KCl) for 30 minutes and three successive changes of the fixative solution (methanol/acetic acid, 3:1). ESCC tissue samples were cut into small pieces in phosphate-buffered saline (PBS), and the interphase nuclei were then prepared following the procedures described above. Metaphase chromosomes and interphase cell nuclei in suspensions were stored at 4°C overnight and then stored at -20°C until use. The nuclear suspensions were dropped onto clean slides and aged at room temperature for 2-3 days prior to the FISH experiments.

### Fluorescence in situ hybridization (FISH)

M-FISH was performed on the metaphase spreads. The 24-color whole chromosome painting (WCP) and arm-specific probes were directly labeled with diethylaminocoumarin (DEAC)-dUTP (PerkinElmer Inc., USA), Green-dUTP (Abbott Molecular, USA), Cy3-dUTP (GE Healthcare, USA) or Alexa594-dUTP (Invitrogen) by degenerate oligonucleotide primed-polymerase chain reaction (DOP-PCR) [39] or were indirectly labeled with Biotin-11-dCTP (Invitrogen). Biotin was then visualized with streptavidin-conjugated Cy5 (Jackson Immunoresearch Laboratories Inc., USA). The 12-color FISH probes were labeled with Green-dUTP, Cy3-dUTP, Alexa594-dUTP and Cy5-dUTP.

Split regions were detected using dual-color break-apart bacterial artificial chromosome (BAC) DNA clone probes, which were labeled with Green-dUTP and Cy3-dUTP by random priming using BioPrime DNA labeling system (Invitrogen). BAC DNA clones were selected according to their descriptions in Ensembl database ( http://www.ensembl.org). BAC DNA clones used to detect splitting of 11q13.3-q13.4 and associated regions included NONSC16D6 (68,072,319-68,278,585, hg18), Cancer_1D11 (69,162,501-69,323,924, hg18), NONSC2E5 (70,236,623-70,391,405, hg18), NONSC3C5 (71,992,715-72,182,751, hg18) and NONSC15F5 (75,107,934-75,273,492, hg18).

The slides for M-FISH and dual-color break-apart FISH analyses were pretreated with RNase A (100 mg/ml in 2 x saline sodium citrate [SSC]) and pepsin (50 mg/ml in 0.01 mol/l HCl). The slides were subsequently denatured in 70% formamide/2 x SSC at 73°C-75°C for 3 minutes, quickly cooled with two rinses of 2 x SSC at 4°C, dehydrated in a gradient series of ethanol (75%, 85% and 100%), and air dried. The labeled probes were precipitated, and redissolved in the hybridization solution (50% formamide, 10% dextran sulfate, 1% Tween-20, 2 x SSC), denatured at 75°C for 8 minutes, and quick-chilled on ice for 2 minutes. Hybridization was performed in a humid chamber at 37°C for 24-48 hours. Post-hybridization washes were performed in 50% formamide/2 x SSC for 15 minutes at 43°C and were performed twice for 3 minutes each in 2 x SSC. The slides were dehydrated in 75%, 85% and 100% ethanol, air dried, counterstained with 40,6-diamidino-2-phenylindole (DAPI) (1 mg/ml) and covered with coverslips.

For 12-color FISH analysis [28], the slides were hybridized twice on metaphase spreads as previously described, which was named two-round FISH. After digital fluorescence image acquisition, coverslips on the slides were removed by dipping in 100% ethanol for 30 min, and washed twice in 100% ethanol for 3 min each time, then air dried, and then the slides could be denatured as the above procedures.

### Microscopy and digital image analysis

FISH images were captured using a Zeiss Axio fluorescence microscope equipped with a cooled charged-coupled device (CCD) camera (Princeton Instruments, USA) or a JAI M4 Plus CCD camera (Metasystems International, Germany). All of the fluorescent images were captured with individual single-band-pass filters specific for visualizing DAPI, DEAC, Green, Cy3, Alexa 594 and Cy5 fluorochromes. Pseudo-color images were constructed and analyzed using MetaMorph (Universal Imaging Corporation, USA) or Metacyte module of Metafer imaging systems (Metasystems International).

### Genomic DNA isolation and oligo array-based comparative genomic hybridization (array-CGH)

Genomic DNA from ESCC cell lines was isolated using DNeasy Blood & Tissue Kit (Qiagen, Germany). Genome-wide copy number studies were then performed using an Agilent 44K oligo array platform (Agilent Technologies, USA), with sex-matched normal human DNA (Promega Corporation, USA) used as the reference. Briefly, 1 μg samples of the tested and reference DNA were digested with AluI and RsaI, and differentially labeled with Cy3-dUTP and Cy5-dUTP using Agilent Genomic DNA Enzymatic Labeling Kit Part Number 5190-0449 (Agilent Technologies), respectively. Then Microcon YM-30 (Millipore) was used to clean up the labeled probes. Tested and reference DNA probes were combined and hybridized onto the microarrays enclosed in Agilent SureHyb-enabled hybridization chambers for 40 hours. After hybridization, slides were washed sequentially and scanned with an Agilent DNA Microarray Scanner. Annotations for the probes were based on UCSC hg18 (NCBI Build 36). CNAs and breakpoint data were analyzed via the Agilent Genomic Workbench Software 5.0, set to use the ADM-2 algorithm, an aberration threshold of 5.0 and an absolute average log_2_ ratio ≥ 0.5.

### Statistical analysis

Statistical analyses were carried out by using the SPSS 17.0 software package. The association between splitting of breakpoint regions and clinico-pathological characteristics were assessed by the χ^2^ test, Fisher’s exact test or Kruskal–Wallis test. Logistic regression analysis was performed to determine the independent predictors of lymph node metastasis. *P* values < 0.05 were considered significant.

## Results

### Copy number alterations

**Figure 1:**
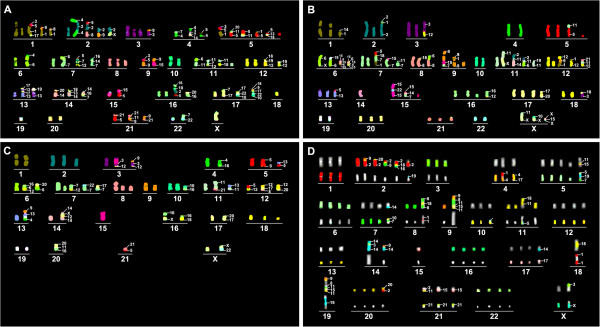
**M-FISH profiling of ESCC cell lines.** A 24-color analysis technique was used for KYSE30 (**A**), KYSE150 (**B**), and YES2 cells (**C**) and 12-color detection was used for KYSE510 cells (**D**).

M-FISH was performed on the metaphase chromosomes of four ESCC cell lines (KYSE30, KYSE150, KYSE510 and YES2). Modal karyotypes of the cell lines are shown in Figure 1. M-FISH karyotypes of two other cell lines KYSE180 [28] and KYSE450 [29] have been previously reported by our laboratory. We found multiple numerical alterations in the six cell line, which exhibited high level of aneuploidy. An overview of CNAs indicated that imbalances occurred throughout the entire genome of the cell lines. Gains were observed at 1p, 1q, 3p, 3q, 4p, 5q, 7p, 8q, 9q, 11q, 14q, 16p, 16q, 17p, 17q, 18p, 19p, 19q, 20q, and 22q. Losses were primarily detected at 3p, 4p, 4q, 6p, 6q, 9p, and 18q.

**Figure 2:**
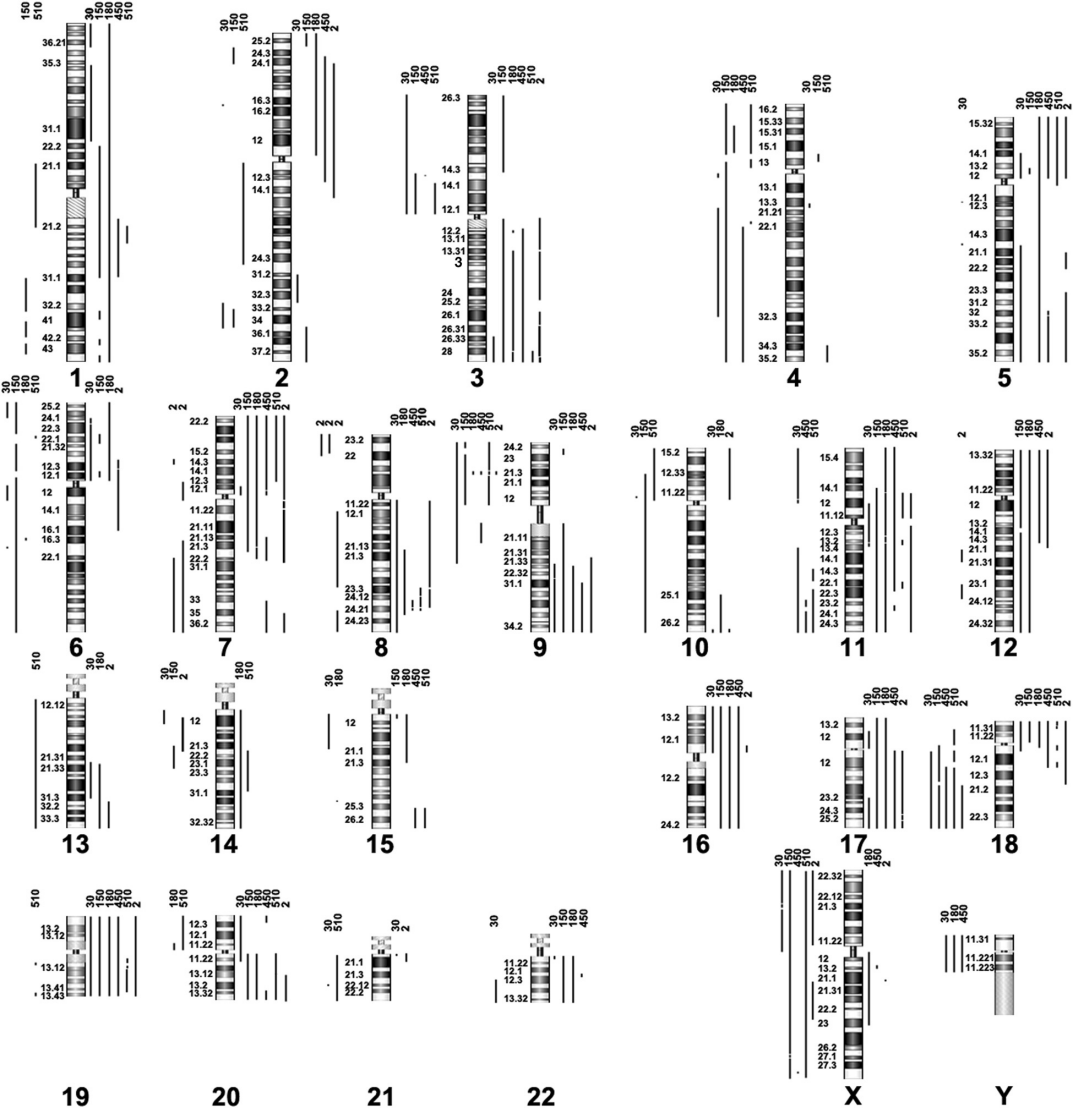
**CNAs and unbalanced breakpoints in six ESCC cell lines detected by array-CGH.** Gains and amplifications are presented as lines on the right side of the chromosomes, while the lines for losses and deletions are on the left side. Unbalanced breakpoints are at the boundaries of CNAs. Numbers on top of the lines are indicated as the cell lines. 30: KYSE30, 150: KYSE150, 180: KYSE180, 450: KYSE450, 510: KYSE510, 2: YES2.

The detail CNAs of these cell lines were detected by array-CGH, and the profiles of gains and losses are shown in Figure 2 and Additional file 1: Table S1. Our results were compared with the data available from Cancer Cell Line Project on the Wellcome Trust Sanger Institute Cosmic website ( http://www.sanger.ac.uk/genetics/CGP/cosmic). Copy number data of KYSE150, KYSE450 and KYSE510 on the website were analyzed using Affymetrix SNP6.0 arrays. Copy number profiles derived from our Agilent 44K platform are very similar to those from the Affymetrix platform. We then compared CNAs among the six cell lines according to the array-CGH data, and frequent gains and losses in at least two cell lines were summarized in Table 1. More gains were found than losses. The results were combined with the data from other 17 ESCC cell lines available on Cosmic website, including KYSE70, KYSE140, KYSE270, KYSE410, KYSE520, Colo-68N, EC-GI-10, HCE-4, TE-1, TE-5, TE-6, TE-8, TE-9, TE-10, TE-11, TE-12 and TE-15. The gains with high frequencies were shown in Additional file 2: Table S2.

**Table 1:** Frequent gains and losses in six ESCC cell lines analyzed by array-CGH

**CNAs**	**Positive No. of cell lines**	**Region**
Gain	≥5	3q26.33-qter, 5p14.1-p11, 7pter-p12.3, 8q24.13-q24.21, 9q31.1-qter, 11p13-p11, 11q11-q13.4, 17q23.3-qter, 18pter-p11, 19, 20q13.32-qter
	3-4	1q21.1-q31.1, 2p24.1-p11, 3q13.31-q26.33, 5pter-p11, 5q31.1-qter, 5q32-qter, 6p21.1-p11, 6p21.31-p11, 7pter-p12.3, 7q11-q22.2, 8q21.2-q24.21, 8q23.3-q24.21, 9q22.1-qter, 10q26.3-qter, 11q11-q22.3, 11q13.4-qter, 12pter-p14.4, 16pter-p11, 16q11-qter, 17p13.1-p11, 17q, 20q11-qter, 20q13.12-qter
	2	1pter-p36.13, 1p35.2-q31.1, 1p22.2-q31.1, 2pter-p25.1, 2p24.2-p11, 2q11-q13, 3q12.1-qter, 5q15-qter, 6p21.33-p21.1, 7q35-qter, 8q11-q24.21, 9q21.33-qter, 11pter-p14.1, 12q14.4-qter, 13q21.32-q31.3, 13q32.1-qter, 14q22.1-q31.1, 15q11, 15q25.3-qter, 16q11-qter, 17pter-p11, 18q11-q12.2, 22q
Loss	≥5	18q21.1-qter
	3-4	3p14.1-p11, 4p15.32-p14, 4q22.1-q32.3, 9pter-p24.1, 9p23-p11, 11q23.3-qter, 18q12.2-q21.1, Xpter-p11, Xq21.1-q23
	2	2q33.1-q35, 3p14.2-p14.1, 4pter-p14.1, 4q21.1-q32.3, 4q22.1-qter, 6pter-p24.1, 6q11-q13, 7q22.2-qter, 8pter-p22, 9pter-p11, 9q11-q21.13, 10p12.33-p11, 11q24.1-qter, 11q22.3-qter, Xq11-qter,

Regions with average log_2_ratio values greater than 1 were defined as amplifications. High-level amplifications (HLAs) and homozygous deletions (HDs) were identified if the absolute average values were no less than 2. According to the positions of HLAs and HDs boundaries, the smallest HLA and HD regions and involved genes among these cell lines were listed in Table 2. HLAs include 7p11 (2/6, 33%), 8q24.21 (2/6, 33%) and 11q13.3-q13.4 (3/6, 50%), harboring several oncogenes, including *EGFR*, *MYC* and *CTTN* (Table 2). Homozygous deletion of 9p21.3, containing tumor suppressor genes *CDKN2A*, *CDKN2B* and *CDKN2B-AS1*, occurred in 67% (4/6) of the cell lines.

**Table 2:** High level amplifications and homozygous deletions in different cell lines

**CNAs**	**Region**^**a**^	**Cell line**	**Start**	**Stop**	**Genes**
HLA	7p11	KYSE30, KYSE450	54572103	57613746	*VSTM2A, SEC61G, EGFR, LANCL2, VOPP1, FKBP9L, ZNF713, MRPS17, GBAS, PSPH, CCT6A, SUMF2, PHKG1, CHCHD2, ZNF479, ZNF716*
	8q24.21	KYSE450, KYSE510	128548643	129216964	*POU5F1B, LOC727677, MYC, PVT1*
	11q13.3-q13.4	KYSE30, KYSE180, KYSE510	68232360	70964483	*MTL5, CPT1A, MRPL21, IGHMBP2, MRGPRD, MRGPRT, TPCN2, MYEOV, CCND1, ORAOV1, FGF19, FGF4, FGF3, ANO1, FADD, PPFIA1, CTTN, SHANK2, DHCR7, NADSYN1, KRTAP5-7, KRTAP5-8, KRTAP5-9, KRTAP5-10, KRTAP5-11*
HD	9p21.3	KYSE180, KYSE450, KYSE510, YES2	21958099	22136626	*CDKN2A, CDKN2B, CDKN2B-AS1*

HLA: high level amplification; HD: homozygous deletion; the Start and Stop positions are annotated by hg18.

^a^ Regions of amplifications or deletions in at least two cell lines.

### Unbalanced breakpoints

Breakpoints were restricted to the boundaries between two adjacent DNA fragments with significantly distinctive log_2_ ratio values, reflecting different copy numbers. Using this scheme, 261 candidate unbalanced breakpoints were identified ( Additional file 3: Table S3). Among these candidates, 39 occurred in the centromeric regions, and the other 224 were present on chromosome arms. Fifty-seven of arm breakpoints were localized in the vicinity of fragile sites. Breakpoints on chromosome arms and copy number status of the regions at both sides of the breakpoints were listed in Additional file 3: Table S3. Cell lines were ranked according to the number of breakpoints, and the top three were KYSE30, KYSE510 and YES2, respectively. This tendency was similar to that in M-FISH results.

### Chromosomal structural aberrations

M-FISH results of four cell lines (KYSE30, KYSE150, KYSE510 and YES2) as well as that of the previously reported two cell lines (KYSE180 and KYSE450) showed that a total of 156 derivative chromosomes resulted from translocations, most of which were unbalanced; only 12.8% (20/156) were reciprocal. Approximately, 35% of the translocation derivative chromosomes were fused at the centromeric regions. Chromosomes 1, 2, 3, 5, 6, 7, 8, 9, 11, 12, 14, 15 and X were frequently rearranged. Combining M-FISH with array-CGH, we further characterized multiple rearrangements present in these cell lines (Table 3). KYSE30 is the cell line with the most complex rearrangements, and array-CGH results have also indicated that much more breakpoints were present in KYSE30 than the other cell lines, which are consistent with M-FISH results.

**Table 3:** Chromosomal structural aberrations analyzed by a combination of M-FISH and array-CGH

**Cell line**	**Chromosomal structural aberrations**
KYSE30	der(1)t(1;8)(p10;q?), der(1;9)(p10;p10), der(1)(3qter → 3q?::5p11 → 5p14.1::1p35.3 → 1q23.2::17p10 → 17p13.1:), der(2;4)(4pter → 4p10::2pter → 2q31.2::4q10 → 4qter), der(2)t(2;9)(p10;q22.3), der(2)t(2;X)(p10;p11), der(2)(5pter → 5p14.1::2p16.2 → 2q31.2::8q10 → 8qter), der(3)t(3;9)(3q10 → qter::9q22.1 → q22.33:), der(4)t(4;5)(q10;p11), der(4)t(4;7)(q13.3;p15.1), der(4)(4pter → 4p10::3q26.33 → 3qter::7p12.1 → 7p11::14q?), der(5)t(1;5)(p36.13;p10), der(5;11)(q10;p10), der(5)(3q? → 3qter::5q10 → 5q15::1q23.2 → 1qter), der(5)(3q26.33 → 3qter::5p14.1 → 5p10::9q22.1 → 9qter), der(5)(5pter → 5p10::11q13.3 → 11q13.4::20q11 → 20q11.23:), der(5)(:5p14.1 → 5p10::13q?::18q11:), der(6)t(4;6)(q?;p24.1), der(7)t(6;7)(p22.3;p14.3), der(5;7)(:5p14.1 → 5p10::7p14.3 → 7q22.1::12p10 → 12pter), der(7)(16pter → 16p11;14q?;7p12.1 → 7q22.1:), der(15)(9q22.3 → 9qter::15q10 → 15qter), der(11)t(6;11)(p10;p12), der(11;18)(p10;p10), der(7;11)(:11q10 → 11q13.3::7q10 → 7q22.1::12q11 → 12q14.1:), der(12;19)(p10;p10), der(12)(12pter → 12q14.1::19p?::18p?), der(13;19)(q10;q10), der(17)(:13q21.32 → 13q31.3::17p13.1 → 17p10::13q21.32 → 13q31.3::17p13.1 → 17p10:), der(14;20)(q10;p10), der(?)(14q?::16p?::7p11 → 7p12.1:), der(14)(18pter → 18p10::13q?::14q10 → 14q31.1:), der(15)t(5;15)(q15;q10), der(16;20)(q10;p10), der(16)(16pter → 16p10::10pter → 10p11.21::2q11 → 2q33.1::?), der(17)(:7p12.1 → 7p11::17q10 → 17qter::17q10 → 17qter), der(17;20)(:17p13.1 → 17p10::22q10 → 22q11.21::10q26.3 → 10qter), der(17)(:18q11::9q22.1 → 9q22.33::17p? → 17p10::22q12.1 → 22q12.3::10p11.21 → 10pter), der(21)t(5;21)(q15;q10), der(21)t(9;21)(q22;q10), der(21)(:9q22.1 → 9q22.33::11q13.3 → 11q13.4::21q10 → 21qter), der(22)t(7;22)(p12.1;q10)
KYSE150	der(1)t(1;14)(q31.1;q1?), der(2)(2pter → 2p24.3::1q32.2 → 1q41::2p24.1 → 2q35:), der(3)t(3;12)(p14.2;q14.1), der(5;11)(q10;p10), der(6)t(6;X)(q?;q27.1), t(15;6;15;6;15)(:15q11::6p22.2 → 6p21.33::15q10 → 15q11::6p12.2 → 6p12.1::15q11:), der(7)t(7;11)(p10;q?), der(7)t(7;15)(q22.1;q11), der(5;7)(1pter → 1p22.2::7q10 → 7q22.1::18q21.1 → 18q21.1::5p10 → 5pter), der(6;8)(p10;q10), der(8)t(8;18)(p22;p11), der(9)(:9p24.1 → 9p23::1q32.3 → 1q41::9p21.1 → 9qter), der(9)(9pter → 9q?::15q11 → 15qter::7q? → 7qter), der(11;19)(q10;p10), der(11)t(11;X)(p14.1;q10), der(11)(:2q35::11p14.1 → 11p10::6p22.2 → 6p21.33:), der(12)t(1;12)(?;q14.1), der(12)(1pter → 1q31.1::9p2? → 9p2?::12p10 → 12pter), t(21;11;12)(?::q?::q? → qter), der(13)t(5;13)(?;q10), der(?)(7q?::14q23.2 → 14qter::15q10 → 15q11:), der(14)(2qter → 2q35::11p1? → 11p1?::14q10 → 14qter), der(15;22)(15q10 → 15qter::22q10 → 22qter::15q10 → 15qter), der(16)t(12;16)(?;pter), der(17;20)(p10;q10), der(18)t(3;18)(p14.2;q10), der(6;19)(q10;p10), der(X)t(11;X)(p14.1;p10), t(X;15;X)(?::15q?::?)
KYSE510	der(2)t(2;8)(q10;q23.3), der(2;9)(q10;q10), der(2)t(2;19)(p10;q1?), der(2)t(2;20)(q33.1;q?), der(2)(2pter → 2p10::18q12.1 → 18q12.2::16p?), der(3;6)(q10;p10), der(4)t(1;4)(p21.1;q10), der(4)t(4;17)(q34.3;?), der(5)(5pter → 5p10::19q13.11 → 19q13.42::11q22.2 → 11q22.3::15q25.3 → 15qter::7pter → 7p12.3), der(7;10)(10q10 → 10qter::7q10 → 7q21.2::14q22.1 → 14q31.1:), der(8)(8pter → 8q12.1::1q21.2 → 1q23.3::18q12.1 → 18q12.2::1q21.2 → 1q23.3:: 8pter → 8q12.1), der(9)(9q10 → 9qter::8q23.3 → 8q24.21::11q13.2 → 11q13.4::15q25.3 → 15qter::11q11 → 11q13.2:), der(10)t(10;19)(q10;q12), der(11)t(11;18)(p10;q11), der(11)(5pter → 5p11::11p13 → 11q14.3::18pter → 18p11.31), der(14)t(9;14)(p11;q10), der(14)(14q?::3q28 → 3qter::14q22.1 → 14q31.1::14q?), der(15)t(15;21)(q10;q?), der(17)t(14;17)(?;q10), der(18)(:1q21.1 → 1q23.3::18pter → 18p10::1q23.3 → 1qter), der(11)(9pter → 9p21.3::19q13.11 → 19q13.42::8pter → 8p21.3::11q13.2 → 11q13.4::15q25.3 → 15qter::11q10 → q?), der(20)t(2;20)(q33.1;q10), der(21)t(15;21)(q25.3;q10), der(21)(:11p13 → 11p11::2p11 → 2p11::21q10 → 21q?), der(X)t(3;X)(p14.1;p22.31)
YES2	der(3)t(3;12)(q29;?), der(3)(9pter → 9p21.3::3q10 → 3q13.31::?), der(4)t(4;16)(q?;p11), der(5)t(5;9)(q?;?), der(5)t(5;13)(p10;q32.1), der(6)t(6;20)(p12.3;q10), der(12)(16pter → 16p11::6p21.1 → 6p12.3::12q10 → 12q21.1:), der(7)t(7;12)(q11.21;q24.11), der(17)t(7;17)(q31.1;p10), der(22)t(7;22)(q11.21;q10), der(10)t(10;16)(p10;p11), der(11)t(11;13)(p10;q32.1), der(11)t(11;21)(pter;?), der(12)t(5;12)(q23.3;p10), der(12)t(12;20)(q21.1;q?), der(12)(12pter → 12p10::17q2?::8q23.3 → 8q24.1:), der(13)(9q21.32 → 9qter::13q10 → 13q32.1::?::4q?), der(14)(14pter → 14q12::X?::12q?::14q22.1 → 14qter), der(15)(15qter → q10::15q10 → 15qter), der(16)t(16;18)(p10;q11), der(16)t(16;X)(q11;?), der(17)(:17q10 → 17q24.3::20q11.21 → 20q?), der(20)(:16p11 → 16p11::11p13 → p11::20p10 → 20pter), der(21)t(5;21)(q10;q21.1), der(X)t(22;X)(q11;p10)

### Recurrent breakpoint regions in ESCC cell lines and primary tumor tissues

**Figure 3:**
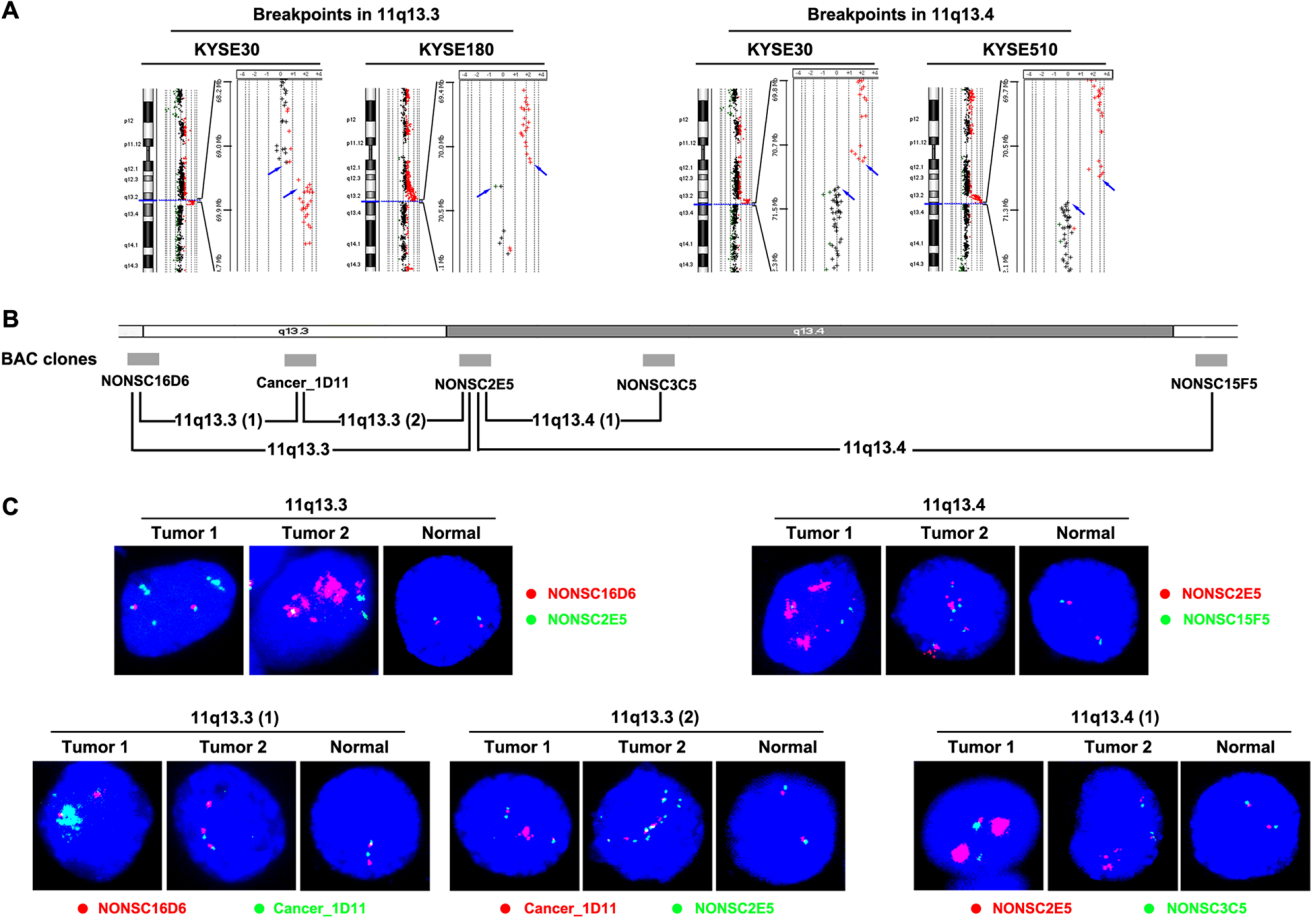
**11q13.3 and 11q13.4 are recurrent breakpoint regions in both the ESCC cell lines and primary tumors.** (**A**) Breakpoints in 11q13.3 and 11q13.4 in the cell lines detected by array-CGH. Breakpoints fall between the probes that displayed significant copy number discrepancies (blue arrows). Amplifications are indicated with red signals. (**B**) The 1-Mb BAC clones that are used in dual-color FISH experiments are shown in the ideogram. Also, 11q13.3 and 11q13.4 are divided into smaller regions according to the positions of BAC DNA clones: 11q13.3(1) (from NONSC16D6 to Cancer_1D11), 11q13.3(2) (from Cancer_1D11 to NONSC2E5) and 11q13.4(1) (from NONSC2E5 to NONSC3C5). (**C**) Detection of 11q13.3-q13.4 breakpoint regions by dual-color FISH in ESCC tumors. BAC DNA probes at two sides of the breakpoint regions are labeled with Cy3-dUTP (red) and Green-dUTP (green). The BAC clones used for each region are listed beside the panel. Two examples of positive tumors are shown for each region. “Tumor 1” and “Tumor 2” samples shown for different regions may not be from the same cases. Most splitting-positive nuclei exhibited amplifications on one side, even high-level amplifications, revealing the breakages between red and green signals. Normal: peripheral blood from normal persons.

Positions of the breakpoints were compared among different cell lines, and further the distances of near breakpoint regions were calculated. Five regions (11q13.4, 9p21.3, 15q25.3, 3q28 and 10q26.3) had breakpoints less than 1 Mb apart, and twelve (11q13.3, 4p13, 11p13, 8q24.21, 2q35, 1q31.1, 21q21.1, 9p21.3, 18q12.2, 3p14.2, 3q12.1-q12.2 and 6p12.3-p12.2) had breakpoints less than 2 Mb apart in different cell lines (Table 4). For example, breakpoints at 11q13.4 were detected in KYSE30 and KYSE510, while breakpoints at 11q13.3 were detected in KYSE30 and KYSE180 (Figure 3A). The three cell lines presented gain of 11q and amplification of 11q13, in which copy numbers of the regions flanking centromere to 11q13 amplicon was higher than the region distal to the amplicon. Losses of the region distal to 11q13 were also found as del(11q13.4-qter) in KYSE30, del(11q14.3-q21) and del(11q22.3-qter) in KYSE510. Translocations of highly amplified regions were also observed (Table 3).

**Table 4:** Recurrent breakpoint regions analyzed by array-CGH in ESCC cell lines

**Region**	**Cell line**	**CN status**^**a**^	**BP Intervals (hg 18) in array-CGH**	**Distance between cell lines**^**b**^	**Genes**^**c**^	**CFSs**
9p21.3 a	KYSE180	Neutral/Del	21958099-21968346	115.1 kb	*C9orf53*^§^, CDKN2A^*^, CDKN2B^†^*, CDKN2B-AS1*^†^	*FRA9C*
	KYSE450	Neutral/Del	21853263-21968346		*MTAP*^§^*, C9orf53*^§^, CDKN2A^*^	
	KYSE510	Loss/Del	21958099-21968346		*C9orf53*^§^, CDKN2A^*^, CDKN2B^†^*, CDKN2B-AS1*^†^	
	YES2	Neutral/Del	21853263-21957548		*MTAP*^§^*, C9orf53*^†^*, CDKN2A*^†^	
15q25.3	KYSE450	Neutral/Gain	86361096-86429254	154.2 kb	*LINC00052*^§^, NTRK3^*^, MRPL46^†^*, MRPS11*^†^	
	KYSE510	Neutral/Gain	86275066-86429254		*LINC00052*^§^, NTRK3^*^, MRPL46^†^*, MRPS11*^†^	
11q13.4	KYSE30	Amp/Loss	70964483-71305189	340.1 kb	*KRTAP5-11*^§^*, FAM86C1, DEFB108B, RNF121*^†^	*FRA11H*
	KYSE510	Amp/Neutral	70964483-71305189		*KRTAP5-11*^§^*, FAM86C1, DEFB108B, RNF121*^†^	
3q28	KYSE180	Gain/Gain	191171376-191222891	517.0 kb	*TP63*^§^, LEPREL1^*^, CLDN1^†^	
	KYSE510	Neutral/Gain	191610761-191688399		*CLDN1*^§^*, CLDN16, TMEM207*^†^*, IL1RAP*^†^	
10q26.3	KYSE30	Neutral/Gain	133045086-133476780	801.8 kb	*TCERG1L*^§^*, PPP2R2D*^†^	
	YES2	Neutral/Gain	133795639-133846905		*BNIP3*^§^, JAKMIP3^*^, DPYSL4^†^	
11q13.3	KYSE30	Amp/Amp	69339391-69569221	1.05 Mb	*FGF3*^§^*, ANO1*^†^*, FADD*^†^	*FRA11H*
	KYSE180	Amp/Amp	70182767-70386856		*CTTN*^§^, SHANK2^*^, DHCR7^†^	
4p13	KYSE150	Amp/Loss	41832777-42109513	1.15 Mb	*SLC30A9*^§^*, BEND4, SHISA3*^†^*, ATP8A1*^†^	
	KYSE510	Neutral/Loss	40955943-41226036		*UCHL1*^§^, LIMCH1^*^, PHOX2B^†^	
11p13	KYSE510	Neutral/Gain	33107818-33136537	1.2 Mb	*TCP11L1*^§^*, PIGCP1*^§^, CSTF3^*^, HIPK3^†^	
	YES2	Neutral/Gain	34278741-34307224		*NAT10*^§^, ABTB2^*^, CAT^†^	
8q24.21	KYSE450	Amp/Neutral	129216964-129574570	1.23 Mb	*FAM84B*^§^*, POU5F1B, MYC*^†^*, PVT1*^†^	
	KYSE510	Amp/Loss	129972316-130159085		*FAM84B*^§^*, POU5F1B, MYC*^†^*, PVT1*^†^	
	YES2	Amp/Neutral	130159144-130451718		far from genes, *PVT1*^§^*, GSDMC*^†^	
2q35	KYSE30	Loss/Neutral	217432472-218386863	1.37 Mb	*DIRC3*^§^*, TNS1, CXCR2P1*^†^	
	KYSE150	Loss/Gain	218517852-218801703		*TNS1*^§^*, CXCR2P1, CXCR2, CXCR1, HMGB1P9, ARPC2*^†^	
1q31.1	KYSE150	Gain/Loss	186080345-186315797	1.40 Mb	*PLA2G4A*^§^, *FAM5C*^†^	*FRA1K*
	KYSE450	Gain/Neutral	184912220-185051701		*PTGS2*^§^*, PLA2G4A*^†^	
21q21.1	KYSE30	Gain/Neutral	17060792-17145790	1.48 Mb	*USP25*^§^*, C21orf34*^§^*, CXADR*^†^	
	YES2	Gain/Neutral	18435266-18540695		*CHODL-AS1*^§^*, CHODL*^†^*, TMPRSS15*^†^	
9p21.3 b	KYSE180	Del/Neutral	21999029-22136626	1.45 Mb	CDKN2A^*^, CDKN2B, CDKN2B-AS1, DMRTA1^†^	*FRA9C*
	KYSE450	Del/Neutral	21980581-21993651		CDKN2A^*^, CDKN2B, CDKN2B-AS1^*^, DMRTA1^†^	
	KYSE510	Del/Loss	22992377-23425976		DMRTA1^*^, ELAVL2^†^	
	YES2	Del/Neutral	21999029-22136626		*CDKN2B, CDKN2B-AS1, DMRTA1*^†^	
18q12.2	KYSE450	Gain/Loss	33583906-33747373	1.64 Mb	*CELF4*^§^*, LOC647946*^†^	*FRA18A*
	KYSE510	Amp/Loss	32107441-32200063		*MOCOS*^§^*, FHOD3, C18orf10*^†^	
3p14.2	KYSE150	Gain/Loss	58573676-58887412	1.69 Mb	*FAM107A*^§^*, FAM3D, C3orf67, FHIT*^†^	*FRA3B*
	KYSE450	Neutral/Del	59933661-60267262		*FHIT*^*#*^	
3q12.1-q12.2	KYSE180	Neutral/Amp	100190484-100877203	1.86 Mb	*TFG*^§^, ABI3BP^*^, IMPG2^†^	
	KYSE450	Neutral/Gain	102009730-102076392		*DCBLD2*^§^*, COL8A1*^†^	
6p12.3-p12.2	KYSE150	Loss/Amp	51932658-52161439	1.9 Mb	*PKHD1*^§^*, IL17A*^†^*, MCM3*^†^	
	YES2	Gain/Gain	50261630-50627364		*DEFB112*^§^*, TFAP2D*^†^	

^a^ Copy number status on the left and right side of the breakpoint regions. CN: copy number, Amp: amplification, Del: deletion.

^b^ The distance between two outermost breakpoints of all the different cell lines.

^c^ These genes are located at or close to breakpoints in each cell line. “^*^”: Obvious breakpoints were detected inside of genes. “^§^” and “^†^”: Genes at the left and right side of the breakpoint regions, respectively. Genes that are not labeled are located in the breakpoint regions, but positions of the exact breakpoints are not determined. “^#^”: Genes with an inside homozygous deletion (HD), and thus might also be disrupted.

Genes which might be interrupted by the recurrent breakpoints in each cell line were listed in Table 4. Ten of these common breakpoint regions were localized in the vicinity of fragile sites. Genes in these cell lines with inner breakpoints included *CDKN2A, LEPREL1, JAKMIP3, LIMCH1, CSTF3, ABTB2, CDKN2B-AS1, FHIT and ABI3BP*. For these genes, one breakpoint could be detected. Small HDs were also observed inside some genes, resulting in two breakpoints, such as *FHIT* gene in KYSE450. Other genes flanking or close to the boundaries might also be influenced by the breakpoints.

To determine whether genomic aberrations found in these cell lines are also present in primary tumors, we first tested a small sample of 15 ESCC tumors by dual-color FISH. This analysis revealed splitting of regions 11q13.3-q13.4, 9p21, 15q25.3 and 3q28, which presented the highest frequency of disruption in the cell lines. Splitting of these regions had occurred in 5, 1, 2 and 3 out of 15 tumors, respectively. We also examined online data of ESCC cell lines. The results showed that both high level amplifications and breakages existed at 67-72 Mb positions in 11q13 (Figure 3). Multiple breakpoints are present in most of the cell lines, revealing these positions may be highly rearranged.

Due to the highest splitting frequency of 11q13.3-q13.4 in the initial 15 cases, we further expanded the sample pool to further characterize splitting of this region in primary ESCC cases (Figures 3B and 3C). Splitting frequencies of 11q13.4 and 11q13.3 were 36.6% (49/134) and 23.4% (32/137), respectively. Overall, breakage of 11q13.3-q13.4 was observed in 58 out of 134 cases (43.3%). Next, we divided the whole 11q13.3-q13.4 region into several parts, including 11q13.3(1) (*CPT1A, MRPL21, IGHMBP2, MRGPRD, MRGPRF, TPCN2, MYEOV*), 11q13.3(2) (*CCND1, ORAOV1, FGF19, FGF4, FGF3, ANO1, FADD, PPFIA1, CTTN* and *SHANK2*), 11q13.4(1) (*DHCR7, NADSYN1, KRTAP5-7, KRTAP5-8, KRTAP5-9, KRTAP5-10, KRTAP5-11, FAM86C1, DEFB108B, RNF121, IL18BP, NUMA1, LRTOMT, FOLR3, FOLR1, FOLR2, INPPL1, PHOX2A* and *CLPB*). Regions of 11q13.3(1), 11q13.3(2), 11q13.4(1), 11q13.3(2)-q13.4 and 11q13.3(2)-q13.4(1) were split in 16.3% (22/135), 8.4% (11/131), 24.1% (32/133), 41.0% (55/134) and 30.0% (39/130) of the primary ESCC tumors, respectively. Almost all of the array-CGH images of the cell lines in Figure 3A and Additional file 4: Figure S1 showed amplification of the region proximal or distal to the breakpoints. Similarly, most of the splitting-positive ESCC tumors examined by FISH presented focal high-level amplification of the region. The majority of breakpoints between NONSC16D6 and Cancer_1D11 were proximal to the amplicon, while most of the breakpoints between Cancer_1D11 and NONSC2E5 as well as those between NONS2E5 and NONSC15F5 were distal to the amplicon (Figure 3C and Additional file 5: Table S4).

### Correlations between split and amplified regions and clinicopathological characteristics

Clinicopathological parameters of each patient were listed in Additional file 6: Table S5, and the relationships between regional splitting events and clinicopathological characteristics were summarized in Table 5. Splitting of 11q13.3-q13.4 was significantly correlated with lymph node metastasis (LNM) (*P* = 0.004) and advanced stages (*P* = 0.004). In LNM-positive group, 54.9% (39/71) of the tumors exhibited splitting compared with 30.2% (19/63) in LNM-negative group. Tumors at stages IIb/III/IV (53.9%, 41/76) showed higher frequencies of splitting than those at stages I/IIa (29.3%, 17/58). Breakpoint regions of 11q13.4, 11q13.4(1), 11q13.3(2)-q13.4(1) and 11q13.3(2)-q13.4 were also associated with LNM and advanced stages (*P* < 0.05, Additional file 7: Table S6). No correlations were observed between splitting in these regions and other clinico-pathological parameters, including gender, age, tumor size, and differentiation (Table 5 and Additional file 7: Table S6). We also tested the relationship between amplification of this region and clinical features. 11q13.3-q13.4 amplifications were defined as the number of FISH signals was at least 3 ( Additional file 5: Table S4). A positive correlation was observed between 11q13.3-q13.4 amplification and LNM (*P* = 0.022) or advanced stages (*P* = 0.039) (Table 5). Amplification of 11q13.3 or 11q13.4 alone was not associated with the parameters ( Additional file 8: Table S7).

**Table 5:** Relationship between splitting and amplification of 11q13.3-q13.4 and clinical features of primary ESCC tumors

**Clinical features**	**Splitting**		**Amplification**	
	**Frequency**	***P *** **value**	**Frequency**	***P *** **value**
Gender				
Male	47.2% (50/106)	0.089 ^a^	86.2% (94/109)	1.000 ^a^
Female	28.6% (8/28)		89.3% (25/28)	
Age				
< 60	47.8% (33/69)	0.274	87.1% (61/70)	1.000 ^a^
≥ 60	38.5% (25/65)		86.6% (58/67)	
Tumor size				
T1, T2	50.0% (10/20)	0.511	85.0% (17/20)	0.728 ^a^
T3, T4	42.1% (48/114)		87.2% (102/117)	
Lymph node metastasis				
N0	30.2% (19/63)	0.004	79.4% (50/63)	0.022 ^a^
N1	54.9% (39/71)		93.2% (69/74)	
Stage				
I, IIa	29.3% (17/58)	0.004	79.3% (46/58)	0.039 ^a^
IIb, III, IV	53.9% (41/76)		92.4% (73/79)	
Differentiation				
G1	40.7% (11/27)	0.762 ^b^	85.2% (23/27)	0.118 ^b^
G2	41.7% (30/72)		91.9% (68/74)	
G3	48.6% (17/35)		77.8% (28/36)	

^a^ Fisher’s test.

^b^ Kruskal–Wallis test.

The *P* value which is not labeled with “a” or “b” is assessed by χ2 test.

In order to create a multivariate model describing the risk for LNM, univariate and multivariate logistic regression analyses were performed with respect to gender, age, tumor size, differentiation status, as well as 11q13.3-q13.4 splitting and amplification. Multivariate analysis indicated that only splitting of 11q13.3-q13.4 was an independent predictor for LNM in ESCC (*P* = 0.026, RR = 2.357, Table 6).

**Table 6:** Logistic regression analyses of the impact of clinico-pathological factors and 11q13.3-q13.4 splitting and amplification on LNM

**Clinical features**	**LNM**		**Univariate analysis**			**Multivariate analysis**						
	**N0**	**N1**	***P *** **value**	**RR (95% CI)**		***P *** **value**^**a**^	**RR (95% CI)**					
Gender							
Male	48	61	0.368	1.466 (0.637-3.374)		-	
Female	15	13					
Age							
< 60	32	38	0.948	0.978 (0.499-1.915)		-	
≥ 60	31	36					
Tumor size							
T1, T2	11	9	0.384	1.528 (0.589-3.964)		-	
T3, T4	52	65					
Differentiation							
G1	16	11	0.295	1.308 (0.792-2.161)		-	
G2	31	43					
G3	16	20					
Splitting status							
Non-splitting	44	32	0.004	2.822 (1.384-5.757)		0.026	2.357 (1.110-5.004)
Splitting	19	39					
Amplification status							
Non-amp	13	5	0.022	3.588 (1.202-10.712)		0.165	2.265 (0.715-7.175)
Amp	50	69					

^a^ The *P* value of each variable which is not significantly correlated with LNM in univariate analysis is indicated with “-” in the multivariate analysis. Multivariate logistic regression analysis is performed using forward procedures.

LNM: lymph node metastasis; RR, relative risk; CI, confidence interval; amp: amplification.

## Discussion

Genomic numerical and structural alterations are common features in ESCC. Our study characterized CNAs, structural aberrations, and recurrent breakpoints in six ESCC cell lines by a combination of M-FISH and array-CGH analyses, which helps provide accurate karyotypes of these cell lines. We further found the correlation between splitting of an amplified region 11q13.3-q13.4 and lymph node metastasis.

Genomic CNAs may influence gene expression through the following mechanisms. A well known mechanism is that gains or losses may result in gene amplifications or deletions, and thus upregulate or downregulate the protein expression [40]. Different situations may occur on genes at the boundaries of gain or loss regions. CNA boundaries inside of the genes usually indicate gene breakage. Gene rearrangements may result from such breakages, leading to the formation of an aberrant gene product [41]. If the CNA boundaries occur in non-coding regions flanking genes, expression may be controlled by proximity to regulatory sequences from other genes. Alternatively, the recurrent breakpoint may indicate loss of a tumor suppressor gene distal to the CNA boundary [42]. Small deletions inside of the genes may result in structural aberrant proteins, truncated proteins, or even loss-of-function proteins. Small amplifications and deletions inside of genes may also indicate gene breakage, and the gene products may also be affected by rearrangements with the partner gene. On the other hand, many recurrent rearrangements occurred at boundaries of the breakpoints, resulting in fusion genes, truncated genes, as well as other structural variants [2]. Therefore, we focused on the breakpoints with CNAs involved in genomic rearrangements and breakpoints mapped to specific sites.

Copy number profiling of ESCC have been analyzed from different studies. Gains involved regions 5p, 7p, 7q, 8q, 11q, 12p, 14q, 16p, 19q, 20q and have been reported in ESCC cell lines by SKY and CGH [35], as well as at least 60% primary tumors by 32K array-CGH [36]. Recently, gains of 19p13.3-q13.43, 11q13.1-q13.4, 20p13-q13.33, 3q24-q29, 22q11.21-q12.1 have been reported [38]. We detected six cell lines, and compared with online database, the high frequency of gains mainly include 3q26.33-qter, 5p14.1-p11, 7pter-p12.3, 8q24.13-q24.21, 9q31.1-qter, 11p13-p11, 11q11-q13.4, 17q23.3-qter, 18pter-p11, 19p, 19q and 20q13.32-qter. Gain of 3q is prevalent in ESCC, and 3q26-qter was found in 76.5% (39/51) primary tumors [43] and 66.7% (4/6) cell lines [44], suggesting cancer-related genes may be present in 3q26-qter. Evidence has been found that *PIK3CA*[35,45], *PRKCI*[46], and *ZNF639*[47] are amplified and overexpressed in ESCC. *PIK3CA*[45] and *PRKCI*[46] are associated with LNM and overexpression of PIK3CA and TFRC are associated with poor prognosis [48]. We found gain of 5p14.1-p11 existed in 83.3% (5/6) cell lines. In the previous study, gain of 5p13 was detected in 10% (3/29) ESCC cell lines. *SKP2* on 5p13 was amplified and overexpressed in 50% (23/46) ESCC tumors, and was associated with LNM and stage. SKP2 overexpression could protect cancer cells from anoikis, which was mediated in part by the phosphoinositidyl 3-kinase-Akt pathway [49]. Gains of 18p11.2-p11.3 and 18p11.3 were also found in 20.7% and 17.2% cell lines by CGH [31] and FISH [50]. *AURKA* at 20q13 encodes a cell cycle-regulated kinase. Yang et al. found that overexpression of AURKA was existed in 85.7% (6/7) cell lines and 93.1% (27/29) tumors [51]. Recent studies have reported that *AURKA* is a direct target of the MAPK pathway [52]. Overexpression of *AURKA* is independently associated with chromosomal instability in colorectal cancer [53], and *AURKA* expression has a prognostic value in ovarian carcinoma [54]. High-level amplifications of 11q13.3-q13.4, 7p11.2, and 8q24.21 have been observed in this study. Amplification of 11q13.3-q13.4 will be discussed later. Amplification of 7p11.2, which harbors an important oncogene *EGFR*, was also found in ESCC from other studies [36]. Amplification and overexpression of *EGFR* gene may play roles in the invasion and progression in cancer [55], and the elevated expression may be an indicator for tumor recurrence and lower survival in head and neck squamous cell carcinoma (HNSCC) [56]. Amplification of *MYC* in 8q24.21 may contribute to the progression of breast cancer [57].

Losses were previously found on 3p, 5q, 8p, 9p and 11q [36], as well as 4p16.3-q35.2, 13q12.11-q34, 18p11.32-q23, and chromosome Y [38]. In our study, losses were observed on 18q12.2-qter, 3p14.1-p11, 4p15.32-p14, 4q22.1-q32.3, 9pter-p24.1, 9p23-p11, 11q23.3-qter, 18q12.2-q21.1, Xpter-p11, Xq21.1-q23 in more than 50% (3/6) of the cell lines, indicating several tumor suppressor genes may be located in these regions. 9p21.3 (*CDKN2A* and *CDKN2B*) is homozygously deleted in some of ESCC [58,59]. It has been found that *CDKN2A* was fused to *IGH* through the translocation t(9;14)(p21;q32) in a pre-B acute lymphoblastic leukemia cell line [60]. The functional study demonstrated that restoring wild-type *CDKN2A* into the gene deleted ESCC cells significantly inhibited cell invasion, suggesting that inactivation of *CDKN2A* may be involved in ESCC invasion [59]. Our array-CGH results confirmed that HD frequency of 9p21.3 was 66.7% (4/6). Interestingly, *CDKN2A* was deleted in only one cell line, and the other three harbored at least one breakpoint inside of *CDKN2A*. For *CDKN2B*, the inside breakpoint was detected in one cell line, while the other three were homozygously deleted.

Recurrent breakpoint regions were detected in at least two cell lines, including 1q31.1, 2q35, 3p14.2, 3q12.1-q12.2, 3q28, 4p13, 4q22.1, 6p12.3-p12.2, 6p22.2-p22.1, 7q22.2-q22.3, 8q24.21, 9p21.3, 10q26.3, 11p13, 11q13.3, 11q13.4, 13q21.32, 15q25.3, 18q12.2 and 21q21.1. Many of these breakpoints were different from those detected by SKY in other ESCC cell lines [35]. The correlation between breakpoints of fusion genes and fragile sites has been emphasized in previous studies. Burrow et al. analyzed 444 pairs of genes involved in cancer-specific recurrent translocations, and found that 52% of the breakpoints in at least one gene of the fusion-gene pairs were localized within the vicinity of a fragile site [61]. Thus, understanding breakpoints near fragile sites may be helpful for further discovering cancer-related gene rearrangements.

11q13 is an important region that presents various aberrations in many malignancies. Gain of 11q13 has previously been described in ESCC [34,36-38,62,63] and other solid tumors, including oral [42], gastric [64], breast [65-67], ovarian [68], prostate [69], bladder [70], laryngeal [71], nasopharyngeal [72], and liver tumors [73] as well as head and neck [74,75] cancer. Gain frequencies of 11q13 varied between studies. The probable target genes that are amplified and/or overexpressed in different cancers have been reported to include *CCND1*[62,76,77], *FGF4*[62,78], *PPFIA1*[67], *CTTN*[62,76] and *ORAOV1*[79]. We observed that 11q13.3-q13.4 was a region with high-level amplification adjacent to the breakpoint boundaries. Breakpoints in 11q13.4 and 11q13.3 were both found in two cell lines, and breakpoints that were identified in 11q13.4 between KYSE30 and KYSE510 were closer to each other. Furthermore, breakpoints in the entire region of 11q13.3-q13.4 were present in more than 40% of ESCC tumors, suggesting that 11q13.3-q13.4 may be a frequently split region in ESCCs.

11q13 is also involved in various chromosomal rearrangements in both hematological malignancies and epithelial carcinomas. The t(11;14)(q13;q32) translocation is associated with 70%-90% of mantle cell lymphomas (MCL) [80,81], a small portion of multiple myeloma (MM) [81,82], acute myeloid leukemia (AML) [83] and other lymphoproliferative disorders [84]. As a result of this translocation, *CCND1* is fused to the enhancer of immunoglobulin heavy chain gene (*IGH*), and thus overexpressed in MCL and MM [81]. *MYEOV*, proximal to *CCND1*, was also overexpressed in a subset of t(11;14)-positive MM cell lines, in which both *MYEOV* and *CCND1* were under the control of *IGH* enhancers due to translocations [85]. There are also other partner genes fused to *CCND1*, including *IGK* involved in t(2;11)(p11;q13) in leukemic small-cell B-non-Hodgkin lymphoma (NHL) [86] and an unknown partner gene in AML with t(5;11)(q35;q13) [87]. 11q13 rearrangements with 6p21, 7q11.2, 1p13 and 5q35 were observed in renal oncocytoma. Jhang et al. demonstrated that CCND1 was overexpressed only in the cases with 11q13 translocation. However, not all of the cases with 11q13 translocations could lead to CCND1 overexpression [88]. Evidence of fusions involving other genes in 11q13 has been reported. *NUMA-RARA* and *RUNX1-MACROD1* were present in the monocytic leukemia with t(11;17)(q13;q21) [89-91] and APL with t(11;21)(q13;q22) [92], respectively. Rearrangement of *LRP5* was found in AML, although the partner gene has not been identified [93]. Most of the above translocations in lymphomas and leukemia are balanced and not complicated, while more complex rearrangements of 11q13 were detected in epithelial carcinomas, including cervical carcinoma cell lines [94], serous ovarian adenocarcinoma [95], hepatocellular carcinoma [96], gastric cancer [97] and oral squamous cell carcinoma (OSCC) [98,99]. Our M-FISH results were much similar to the observations in these carcinomas. We found that chromosome 11 was frequently rearranged, especially in KYSE30, KYSE150 and KYSE510. In each cell line, five to six derivative chromosomes associated with chromosome 11 were easily found, and some complex derivative chromosomes involved q13 band of the chromosome. Genes involved in breakpoints of these rearrangements remain to be clarified.

The current array-CGH profiling enabled us to set the boundaries of 11q13 amplicons in ESCC cell lines. We observed that multiple breakpoints existed in high level amplification regions involving 11q13.3 were located in 67-72 Mb position in three ESCC cell lines we detected and ten online cell lines, which is similar to the amplification peak in HNSCC [75]. The mechanisms for formation of several amplicons have been well described by a model of breakage-fusion-bridge (BFB) cycle. According to this model, the formation of amplicons is initiated by distal DNA breakages at fragile sites. During DNA replication, a dicentric chromosome with an inverted duplication may be resulted from the sister chromatid fusion (SCF). Breakage-fusion-bridge cycle may continue when another break between two centromeres occurs. The cycle may be then stabilized by a telomere or by translocation [42,98,100-102]. Albertson suggested that amplicon boundaries might also be set by selection for overexpressed genes in the amplicons, or by selection against expression changes of genes outside of amplified regions induced by CNAs [101,103]. 11q13 harbors three fragile sites, *FRA11A*, *FRA11H* and *FRA11F*[104]. *FRA11A* is a rare fragile site, while *FRA11H* and *FRA11F* are common fragile sites. *FRA11A* is located between *RIN1* (11q13.2) and *CCND1* (11q13.3) [98]. *FRA11H* is positioned at 11q13, but the exact location still needs to be characterized. *FRA11F* is located between the BAC clones of RP11-281H14 and RP11-841F15 in 11q14.2 [42]. Reshmi et al. found that OSCC cell lines with complex 11q rearrangements were affected by *FRA11F*, and gene amplifications in 11q13 region in OSCC cell lines may be initiated by breakage at *FRA11F*[42]. Shuster et al. demonstrated that breakages at *FRA11A* between RIN1 and CCND1 may promote the BFB cycles [98]. They also found the involvement of *FRA11H* in some OSCC cases with amplifications of genes in 11q13 [42,105]. In the present study, distal boundaries of amplicons in the majority of ESCC cell lines and primary tumors with 11q13 amplification were clustered within 67-72 Mb region of 11q13.3, which may involve *FRA11H* breakages for these cases. Another breakpoint was observed at *NAALAD2* gene in KYSE510, and it was located within *FRA11F*. In addition, breakpoints proximal of 11q13 amplicons in KYSE180, KYSE510 and five online cell lines were located in *FRA11A* region in 11q13.3, while the proximal breakpoints in KYSE30 and other online cell lines were distal to *FRA11A* or in *FRA11H*. In the tested ESCC tumors, the majority of breakpoints in 11q13.3(1) were proximal to the amplicons, and most of those in 11q13.3(2) and 11q13.4 were distal to the amplicons. Thus, we speculate that initial distal breakages may primarily occur at *FRA11H*, and the process may involve *FRA11F* in some cases. *FRA11A* or *FRA11H* may contribute to setting amplicon boundaries by promoting subsequent steps of BFB cycle. Concerning the presentation of multiple breakpoint boundaries in some of ESCC cell lines and primary tumors with high-level amplification of 11q13, several cycles of random breakages may be undergone.

In the current study, we have noticed that copy numbers of the regions from centromere to boundaries at initial breaks were higher than those of the regions distal to breakpoints in most of ESCC cell lines with 11q13 amplifications. Gains of proximal regions, losses of distal regions, intrachromosomal or interchromosomal rearrangements of 11q13 have been found in the cell lines or primary tumors of human cancer and demonstrated to be indicators of BFB cycle [98,101]. At the end of BFB cycles, distal breakpoints of 11q13.3-q13.4 amplicon may undergo intrachromosomal rearrangements or translocating to other chromosomes, which may affect genes at distal boundaries through forming intragenic rearrangements or fusing to other genes. Notably, most of 11q13.3-q13.4 splitting cell lines according to our and online array-CGH data showed high-level amplification of 11q13 proximal or distal to the breakpoints in ESCC cell lines and primary tumors. Moreover, amplicons involving intrachromosomal or interchromosomal rearrangements have also been detected. Thus, recurrent breakage at 11q13.3-q13.4 may reflect the following aspects. On one hand, genes between two BACs flanking the regions may be amplified, with proximal gain and gene overexpression. On the other hand, breakages between two BACs and thus rearrangements of genes at the amplicon boundaries may also dysregulate expression of these genes.

The relationship between gain of 11q13 and LNM or prognosis have been analyzed and discussed in several studies. However, contrary opinions still exist. Tada et al. conducted CGH on 36 ESCC specimens, and demonstrated that gain of 11q13 did not occur at a significantly different rate between LNM and non-LNM groups [106]. Genes located in 11q13 were analyzed. Amplification of *CTTN* was correlated with LNM, while no significant association was found between *CCND1* amplification and LNM [76], however, predicting *CCND1* amplification using plasma DNA may be an independent prognostic factor in ESCCs [107]. Komatsu et al. found that overexpression of *ORAOV1* showed a significant association with LNM and stages. Gain of 11q13.2 was determined to be an independent prognostic factor for predicting poor outcome, and amplification of *CPT1A* in 11q13.2 was correlated with shorter overall survival in ESCC. Here, we found the correlation between 11q13.3-q13.4 amplification and LNM as well as advanced stages. The relationship between gene status in 11q13 and LNM has also been evaluated in other cancers. Amplification of 11q13 DNA is associated with lymph node involvement in HNSCC [108]. *CCND1* amplification and overexpression are significantly associated with LNM and survival in OSCC [109]. Another study confirmed amplifications of 11 genes in 11q13, and found two amplification cores, including core 1 (*TPCN2* and *MYEOV*), and core 2 (from *CCND1* to *CTTN*). Amplification of *CTTN* (core 2) and/or *TPCN2*/*MYEOV* (core 1) was further demonstrated to be associated with LNM in OSCC [110]. However, Huang et al. reported that there was no correlation between LNM and amplification or expression of the tested genes in 11q13 in OSCC [111]. Fortin et al. also found 11q13 amplifications not appear to be a reliable marker for subclinical LNM prediction in oral and oropharyngeal carcinomas [112]. A study by Xia et al. indicated that amplifications of *ORAOV1* and *CTTN* are indicated to be associated with LNM [113]. In the breast cancer, *PPFIA1* is coamplified with *CCND1*, which is significantly associated with high-grade phenotype but not tumor stage or nodal stage [67].

In this report, we demonstrated that both of 11q13.3-q13.4 splitting and amplification are significantly correlated with LNM and advanced stages, indicating that breakage and amplification of this region may play important roles in the tumor progression. According to multivariate logistic regression analysis, however, it was splitting rather than amplification that could be an independent predictor for the higher tendency of metastasis. In addition, two smaller regions 11q13.4(1) (*SHANK2, DHCR7, NADSYN1, KRTAP5-7, KRTAP5-8, KRTAP5-9, KRTAP5-10, KRTAP5-11, FAM86C1, RNF121, IL18BP, NUMA1, LRTOMT, FOLR3, FOLR1, FOLR2, INPPL1, PHOX2A* and *CLPB*) and 11q13.3(2)-q13.4(1) (*CCND1, ORAOV1, FGF19, FGF4, FGF3, ANO1, FADD, PPFIA1, CTTN*, *SHANK2, DHCR7, NADSYN1, KRTAP5-7, KRTAP5-8, KRTAP5-9, KRTAP5-10, KRTAP5-11, FAM86C1, RNF121, IL18BP, NUMA1, LRTOMT, FOLR3, FOLR1, FOLR2, INPPL1, PHOX2A* and *CLPB*) and were also associated with these parameters. However, no significant difference was found for 11q13.3(1) (*CPT1A, MRPL21, IGHMBP2, MRGPRD, MRGPRT, TPCN2, MYEOV*). These results suggested that genes located in 11q13.3(2) and 11q13.4(1) may play more important roles in LNM than 11q13.3(1). It will be interesting to further investigate gene and protein alterations caused by genomic breakages. Since the exact breakpoint locations may be distinctive in different cases, further studies will be focused on determining which of these genes were rearranged or disrupted in specific cases, identifying possible rearranged forms and roles of these alterations may play in ESCC development.

## Conclusions

Our data provide detailed information on chromosomal and genomic aberrations present in six ESCC cell lines. Using a combination of M-FISH and array-CGH enabled us to produce more accurate karyotypes, which will help to determine appropriate applications of these cell lines for cytogenetic and molecular biological studies. The recurrent genomic breakpoints present in both the cell lines and primary tumors may help to identify aberrant genes associated with the development and progression of ESCC.

## Competing interests

There was no conflict of interest in this study.

## Authors' contributions

HJJ carried out M-FISH experiments, participated in the data analyses, and draft the manuscript. SZZ carried out array-CGH experiments. ZZX organized clinico-pathological information. ZY participated in the design of the study. GT carried out part of the FISH experiments. LCX carried out part of the FISH experiments. ZT performed some statistical analysis. CY performed some statistical analysis. DJT provided FISH probe templates (BAC-DNA) and gave experimental suggestions. FSB provided the statistical analysis suggestion. ZQM gave experimental design suggestions. WMR conceived of the study, and participated in its design and coordination and helped to draft the manuscript. All authors have read and approved the final manuscript.

## Pre-publication history

The pre-publication history for this paper can be accessed here:

http://www.biomedcentral.com/1471-2407/12/367/prepub

## Supplementary Material

Additional file 1 **Table S1.** Genomic copy number alterations in ESCC cell lines detected by array-CGH. Click here for file

Additional file 2 **Table S2.** Regions of gain and the frequencies in the ESCC cell lines. Click here for file

Additional file 3 **Table S3.** Unbalanced breakpoints analyzed according to the copy number alterations. Click here for file

Additional file 4 **Figure S1.** High level amplifications and breakages of 11q13 in ten ESCC cell lines. (A) KYSE140, (B) KYSE410, (C) EC-GI-10, (D) HCE-4, (E) TE-6, (F) TE-8, (G) TE-9, (H) TE-10, (I) TE-11, (J) TE-15. Amplifications are mainly located within 67-72 Mb position. The smallest amplification region is similar to previously reported. More than two breakpoints are present in most of the cell lines, revealing these positions may be highly rearranged. (EPS 3433 kb) Click here for file

Additional file 5 **Table S4.** Splitting and amplification of 11q13.3-q13.4 in the primary ESCC tumors by FISH analyses. Click here for file

Additional file 6 **Table S5.** Clinico-pathological parameters of the examined ESCC patients. Click here for file

Additional file 7 **Table S6.** Relationship between region splittings and clinico-pathological features of ESCC. Click here for file

Additional file 8 **Table S8.** Relationship between region amplifications and clinico-pathological features of ESCC. Click here for file

## References

[B1] Frohling S Dohner H Chromosomal abnormalities in cancer N Engl J Med 2008 359 7 722 734 10.1056/NEJMra0803109 18703475

[B2] Ritz A Paris PL Ittmann MM Collins C Raphael BJ Detection of recurrent rearrangement breakpoints from copy number data BMC Bioinformatics 2011 12 114 10.1186/1471-2105-12-114 21510904 PMC3112242

[B3] Mitelman F Johansson B Mertens F The impact of translocations and gene fusions on cancer causation Nat Rev Cancer 2007 7 4 233 245 10.1038/nrc2091 17361217

[B4] Edwards PA Fusion genes and chromosome translocations in the common epithelial cancers J Pathol 2010 220 2 244 254 19921709 10.1002/path.2632

[B5] Clark JP Cooper CS ETS gene fusions in prostate cancer Nat Rev Urol 2009 6 8 429 439 10.1038/nrurol.2009.127 19657377

[B6] Berger MF Lawrence MS Demichelis F Drier Y Cibulskis K Sivachenko AY Sboner A Esgueva R Pflueger D Sougnez C The genomic complexity of primary human prostate cancer Nature 2011 470 7333 214 220 10.1038/nature09744 21307934 PMC3075885

[B7] Stephens PJ McBride DJ Lin ML Varela I Pleasance ED Simpson JT Stebbings LA Leroy C Edkins S Mudie LJ Complex landscapes of somatic rearrangement in human breast cancer genomes Nature 2009 462 7276 1005 1010 10.1038/nature08645 20033038 PMC3398135

[B8] Inaki K Hillmer AM Ukil L Yao F Woo XY Vardy LA Zawack KF Lee CW Ariyaratne PN Chan YS Transcriptional consequences of genomic structural aberrations in breast cancer Genome Res 2011 21 5 676 687 10.1101/gr.113225.110 21467264 PMC3083084

[B9] Kohno T Ichikawa H Totoki Y Yasuda K Hiramoto M Nammo T Sakamoto H Tsuta K Furuta K Shimada Y KIF5B-RET fusions in lung adenocarcinoma Nat Med 2012 18 3 375 377 10.1038/nm.2644 22327624 PMC6430196

[B10] Ju YS Lee WC Shin JY Lee S Bleazard T Won JK Kim YT Kim JI Kang JH Seo JS A transforming KIF5B and RET gene fusion in lung adenocarcinoma revealed from whole-genome and transcriptome sequencing Genome Res 2012 22 3 436 445 10.1101/gr.133645.111 22194472 PMC3290779

[B11] Bass AJ Lawrence MS Brace LE Ramos AH Drier Y Cibulskis K Sougnez C Voet D Saksena G Sivachenko A Genomic sequencing of colorectal adenocarcinomas identifies a recurrent VTI1A-TCF7L2 fusion Nat Genet 2011 43 10 964 968 10.1038/ng.936 21892161 PMC3802528

[B12] Palanisamy N Ateeq B Kalyana-Sundaram S Pflueger D Ramnarayanan K Shankar S Han B Cao Q Cao X Suleman K Rearrangements of the RAF kinase pathway in prostate cancer, gastric cancer and melanoma Nat Med 2010 16 7 793 798 10.1038/nm.2166 20526349 PMC2903732

[B13] Stransky N Egloff AM Tward AD Kostic AD Cibulskis K Sivachenko A Kryukov GV Lawrence MS Sougnez C McKenna A The mutational landscape of head and neck squamous cell carcinoma Science 2011 333 6046 1157 1160 10.1126/science.1208130 21798893 PMC3415217

[B14] Totoki Y Tatsuno K Yamamoto S Arai Y Hosoda F Ishikawa S Tsutsumi S Sonoda K Totsuka H Shirakihara T High-resolution characterization of a hepatocellular carcinoma genome Nat Genet 2011 43 5 464 469 10.1038/ng.804 21499249

[B15] Tomlins SA Rhodes DR Perner S Dhanasekaran SM Mehra R Sun XW Varambally S Cao X Tchinda J Kuefer R Recurrent fusion of TMPRSS2 and ETS transcription factor genes in prostate cancer Science 2005 310 5748 644 648 10.1126/science.1117679 16254181

[B16] Speicher MR Gwyn Ballard S, Ward DC: Karyotyping human chromosomes by combinatorial multi-fluor FISH Nat Genet 1996 12 4 368 375 10.1038/ng0496-368 8630489

[B17] Schrock E du Manoir S Veldman T Schoell B Wienberg J Ferguson-Smith MA Ning Y Ledbetter DH Bar-Am I Soenksen D Multicolor spectral karyotyping of human chromosomes Science 1996 273 5274 494 497 10.1126/science.273.5274.494 8662537

[B18] Pinkel D Segraves R Sudar D Clark S Poole I Kowbel D Collins C Kuo WL Chen C Zhai Y High resolution analysis of DNA copy number variation using comparative genomic hybridization to microarrays Nat Genet 1998 20 2 207 211 10.1038/2524 9771718

[B19] Speicher MR Carter NP The new cytogenetics: blurring the boundaries with molecular biology Nat Rev Genet 2005 6 10 782 792 10.1038/nrg1692 16145555

[B20] Camps J Grade M Nguyen QT Hormann P Becker S Hummon AB Rodriguez V Chandrasekharappa S Chen Y Difilippantonio MJ Chromosomal breakpoints in primary colon cancer cluster at sites of structural variants in the genome Cancer Res 2008 68 5 1284 1295 10.1158/0008-5472.CAN-07-2864 18316590 PMC4729303

[B21] Mao X James SY Yanez-Munoz RJ Chaplin T Molloy G Oliver RT Young BD Lu YJ Rapid high-resolution karyotyping with precise identification of chromosome breakpoints Genes Chromosomes Cancer 2007 46 7 675 683 10.1002/gcc.20452 17431877

[B22] Watson SK DeLeeuw RJ Horsman DE Squire JA Lam WL Cytogenetically balanced translocations are associated with focal copy number alterations Hum Genet 2007 120 6 795 805 10.1007/s00439-006-0251-9 17051368

[B23] Kawamata N Ogawa S Zimmermann M Niebuhr B Stocking C Sanada M Hemminki K Yamatomo G Nannya Y Koehler R Cloning of genes involved in chromosomal translocations by high-resolution single nucleotide polymorphism genomic microarray Proc Natl Acad Sci U S A 2008 105 33 11921 11926 10.1073/pnas.0711039105 18697940 PMC2575257

[B24] Persson F Winnes M Andren Y Wedell B Dahlenfors R Asp J Mark J Enlund F Stenman G High-resolution array CGH analysis of salivary gland tumors reveals fusion and amplification of the FGFR1 and PLAG1 genes in ring chromosomes Oncogene 2008 27 21 3072 3080 10.1038/sj.onc.1210961 18059337

[B25] Howarth KD Blood KA Ng BL Beavis JC Chua Y Cooke SL Raby S Ichimura K Collins VP Carter NP Array painting reveals a high frequency of balanced translocations in breast cancer cell lines that break in cancer-relevant genes Oncogene 2008 27 23 3345 3359 10.1038/sj.onc.1210993 18084325 PMC2423006

[B26] Jemal A Bray F Center MM Ferlay J Ward E Forman D Global cancer statistics CA Cancer J Clin 2011 61 2 69 90 10.3322/caac.20107 21296855

[B27] Parkin DM Bray FI Devesa SS Cancer burden in the year 2000. The global picture Eur J Cancer 2001 37 Suppl 8 S4 66 11602373 10.1016/s0959-8049(01)00267-2

[B28] Wu YP Yang YL Yang GZ Wang XY Luo ML Zhang Y Feng YB Xu X Han YL Cai Y Identification of chromosome aberrations in esophageal cancer cell line KYSE180 by multicolor fluorescence in situ hybridization Cancer Genet Cytogenet 2006 170 2 102 107 10.1016/j.cancergencyto.2006.05.006 17011979

[B29] Wu YP Yang YL Han YL Xu X Cai Y Yang GZ Wang XY Zhan QM Wu M Wang MR Identification of complex chromosome abnormalities in esophageal carcinoma cells KYSE450 by multicolor fluorescence in situ hybridization Shijie Huaren Xiaohua Zazhi 2006 14 8 747 751

[B30] Yang Y Chu J Wu Y Luo M Xu X Han Y Cai Y Zhan Q Wang M Chromosome analysis of esophageal squamous cell carcinoma cell line KYSE 410-4 by repetitive multicolor fluorescence in situ hybridization J Genet Genomics 2008 35 1 11 16 10.1016/S1673-8527(08)60002-8 18222404

[B31] Pimkhaokham A Shimada Y Fukuda Y Kurihara N Imoto I Yang ZQ Imamura M Nakamura Y Amagasa T Inazawa J Nonrandom chromosomal imbalances in esophageal squamous cell carcinoma cell lines: possible involvement of the ATF3 and CENPF genes in the 1q32 amplicon Jpn J Cancer Res 2000 91 11 1126 1133 10.1111/j.1349-7006.2000.tb00895.x 11092977 PMC5926289

[B32] Wei F Ni J Wu SS Liu H Xu X Han YL Cai Y Zhang JW Chen XJ Pang H Cytogenetic studies of esophageal squamous cell carcinomas in the northern Chinese population by comparative genomic hybridization Cancer Genet Cytogenet 2002 138 1 38 43 10.1016/S0165-4608(02)00586-1 12419583

[B33] Qin YR Wang LD Fan ZM Kwong D Guan XY Comparative genomic hybridization analysis of genetic aberrations associated with development of esophageal squamous cell carcinoma in Henan, China World J Gastroenterol 2008 14 12 1828 1835 10.3748/wjg.14.1828 18350619 PMC2700406

[B34] Sakai N Kajiyama Y Iwanuma Y Tomita N Amano T Isayama F Ouchi K Tsurumaru M Study of abnormal chromosome regions in esophageal squamous cell carcinoma by comparative genomic hybridization: relationship of lymph node metastasis and distant metastasis to selected abnormal regions Dis Esophagus 2010 23 5 415 421 19930403 10.1111/j.1442-2050.2009.01026.x

[B35] Yen CC Chen YJ Lu KH Hsia JY Chen JT Hu CP Chen PM Liu JH Chiou TJ Wang WS Genotypic analysis of esophageal squamous cell carcinoma by molecular cytogenetics and real-time quantitative polymerase chain reaction Int J Oncol 2003 23 4 871 881 12963965

[B36] Carneiro A Isinger A Karlsson A Johansson J Jonsson G Bendahl PO Falkenback D Halvarsson B Nilbert M Prognostic impact of array-based genomic profiles in esophageal squamous cell cancer BMC Cancer 2008 8 98 10.1186/1471-2407-8-98 18405350 PMC2374796

[B37] Hu N Wang C Ng D Clifford R Yang HH Tang ZZ Wang QH Han XY Giffen C Goldstein AM Genomic characterization of esophageal squamous cell carcinoma from a high-risk population in China Cancer Res 2009 69 14 5908 5917 10.1158/0008-5472.CAN-08-4622 19584285 PMC2734334

[B38] Shi ZZ Liang JW Zhan T Wang BS Lin DC Liu SG Hao JJ Yang H Zhang Y Zhan QM Genomic alterations with impact on survival in esophageal squamous cell carcinoma identified by array comparative genomic hybridization Genes Chromosomes Cancer 2011 50 7 518 526 10.1002/gcc.20875 21484929

[B39] Telenius H Carter NP Bebb CE Nordenskjold M Ponder BA Tunnacliffe A Degenerate oligonucleotide-primed PCR: general amplification of target DNA by a single degenerate primer Genomics 1992 13 3 718 725 10.1016/0888-7543(92)90147-K 1639399

[B40] Albertson DG Collins C McCormick F Gray JW Chromosome aberrations in solid tumors Nat Genet 2003 34 4 369 376 10.1038/ng1215 12923544

[B41] Nambiar M Kari V Raghavan SC Chromosomal translocations in cancer Biochim Biophys Acta 2008 1786 2 139 152 18718509 10.1016/j.bbcan.2008.07.005

[B42] Reshmi SC Huang X Schoppy DW Black RC Saunders WS Smith DI Gollin SM Relationship between FRA11F and 11q13 gene amplification in oral cancer Genes Chromosomes Cancer 2007 46 2 143 154 10.1002/gcc.20394 17099871

[B43] Ueno T Tangoku A Yoshino S Abe T Toshimitsu H Furuya T Kawauchi S Oga A Oka M Sasaki K Gain of 5p15 detected by comparative genomic hybridization as an independent marker of poor prognosis in patients with esophageal squamous cell carcinoma Clin Cancer Res 2002 8 2 526 533 11839673

[B44] Tada K Oka M Hayashi H Tangoku A Oga A Sasaki K Cytogenetic analysis of esophageal squamous cell carcinoma cell lines by comparative genomic hybridization: relationship of cytogenetic aberrations to in vitro cell growth Cancer Genet Cytogenet 2000 117 2 108 112 10.1016/S0165-4608(99)00160-0 10704679

[B45] Akagi I Miyashita M Makino H Nomura T Hagiwara N Takahashi K Cho K Mishima T Ishibashi O Ushijima T Overexpression of PIK3CA is associated with lymph node metastasis in esophageal squamous cell carcinoma Int J Oncol 2009 34 3 767 775 19212681 10.3892/ijo_00000202

[B46] Yang YL Chu JY Luo ML Wu YP Zhang Y Feng YB Shi ZZ Xu X Han YL Cai Y Amplification of PRKCI, located in 3q26, is associated with lymph node metastasis in esophageal squamous cell carcinoma Genes Chromosomes Cancer 2008 47 2 127 136 10.1002/gcc.20514 17990328

[B47] Imoto I Yuki Y Sonoda I Ito T Shimada Y Imamura M Inazawa J Identification of ZASC1 encoding a Kruppel-like zinc finger protein as a novel target for 3q26 amplification in esophageal squamous cell carcinomas Cancer Res 2003 63 18 5691 5696 14522885

[B48] Wada S Noguchi T Takeno S Kawahara K PIK3CA and TFRC located in 3q are new prognostic factors in esophageal squamous cell carcinoma Ann Surg Oncol 2006 13 7 961 966 10.1245/ASO.2006.08.006 16788758

[B49] Wang XC Wu YP Ye B Lin DC Feng YB Zhang ZQ Xu X Han YL Cai Y Dong JT Suppression of anoikis by SKP2 amplification and overexpression promotes metastasis of esophageal squamous cell carcinoma Mol Cancer Res 2009 7 1 12 22 10.1158/1541-7786.MCR-08-0092 19147533

[B50] Nakakuki K Imoto I Pimkhaokham A Fukuda Y Shimada Y Imamura M Amagasa T Inazawa J Novel targets for the 18p11.3 amplification frequently observed in esophageal squamous cell carcinomas Carcinogenesis 2002 23 1 19 24 10.1093/carcin/23.1.19 11756219

[B51] Yang SB Zhou XB Zhu HX Quan LP Bai JF He J Gao YN Cheng SJ Xu NZ Amplification and overexpression of Aurora-A in esophageal squamous cell carcinoma Oncol Rep 2007 17 5 1083 1088 17390048

[B52] Furukawa T Kanai N Shiwaku HO Soga N Uehara A Horii A AURKA is one of the downstream targets of MAPK1/ERK2 in pancreatic cancer Oncogene 2006 25 35 4831 4839 10.1038/sj.onc.1209494 16532023

[B53] Baba Y Nosho K Shima K Irahara N Kure S Toyoda S Kirkner GJ Goel A Fuchs CS Ogino S Aurora-A expression is independently associated with chromosomal instability in colorectal cancer Neoplasia 2009 11 5 418 425 19412426 10.1593/neo.09154 PMC2671854

[B54] Mendiola M Barriuso J Marino-Enriquez A Redondo A Dominguez-Caceres A Hernandez-Cortes G Perez-Fernandez E Sanchez-Navarro I Vara JA Suarez A Aurora kinases as prognostic biomarkers in ovarian carcinoma Hum Pathol 2009 40 5 631 638 10.1016/j.humpath.2008.10.011 19157502

[B55] Miyai K Yamamoto S Asano T Tamai S Matsubara O Tsuda H Protein overexpression and gene amplification of epidermal growth factor receptor in adult testicular germ cell tumors: potential role in tumor progression Cancer Sci 2010 101 9 1970 1976 10.1111/j.1349-7006.2010.01638.x 20608935 PMC11159324

[B56] Rubin Grandis J Melhem MF Gooding WE Day R Holst VA Wagener MM Drenning SD Tweardy DJ Levels of TGF-alpha and EGFR protein in head and neck squamous cell carcinoma and patient survival J Natl Cancer Inst 1998 90 11 824 832 10.1093/jnci/90.11.824 9625170

[B57] Singhi AD Cimino-Mathews A Jenkins RB Lan F Fink SR Nassar H Vang R Fetting JH Hicks J Sukumar S MYC gene amplification is often acquired in lethal distant breast cancer metastases of unamplified primary tumors Mod Pathol 2012 25 3 378 387 10.1038/modpathol.2011.171 22056952 PMC3276715

[B58] Maesawa C Tamura G Nishizuka S Ogasawara S Ishida K Terashima M Sakata K Sato N Saito K Satodate R Inactivation of the CDKN2 gene by homozygous deletion and de novo methylation is associated with advanced stage esophageal squamous cell carcinoma Cancer Res 1996 56 17 3875 3878 8752149

[B59] Kim DH Muto M Kuwahara Y Nakanishi Y Watanabe H Aoyagi K Ogawa K Yoshida T Sasaki H Array-based comparative genomic hybridization of circulating esophageal tumor cells Oncol Rep 2006 16 5 1053 1059 17016592

[B60] Urashima M Hoshi Y Sugimoto Y Kaihara C Matsuzaki M Chauhan D Ogata A Teoh G DeCaprio JA Anderson KC A novel pre-B acute lymphoblastic leukemia cell line with chromosomal translocation between p16(INK4A)/p15(INK4B) tumor suppressor and immunoglobulin heavy chain genes: TGFbeta/IL-7 inhibitory signaling mechanism Leukemia 1996 10 10 1576 1583 8847892

[B61] Burrow AA Williams LE Pierce LC Wang YH Over half of breakpoints in gene pairs involved in cancer-specific recurrent translocations are mapped to human chromosomal fragile sites BMC Genomics 2009 10 59 10.1186/1471-2164-10-59 19183484 PMC2642838

[B62] Arai H Ueno T Tangoku A Yoshino S Abe T Kawauchi S Oga A Furuya T Oka M Sasaki K Detection of amplified oncogenes by genome DNA microarrays in human primary esophageal squamous cell carcinoma: comparison with conventional comparative genomic hybridization analysis Cancer Genet Cytogenet 2003 146 1 16 21 10.1016/S0165-4608(03)00106-7 14499691

[B63] Hirasaki S Noguchi T Mimori K Onuki J Morita K Inoue H Sugihara K Mori M Hirano T BAC clones related to prognosis in patients with esophageal squamous carcinoma: an array comparative genomic hybridization study Oncologist 2007 12 4 406 417 10.1634/theoncologist.12-4-406 17470683

[B64] Kuroda A Tsukamoto Y Nguyen LT Noguchi T Takeuchi I Uchida M Uchida T Hijiya N Nakada C Okimoto T Genomic profiling of submucosal-invasive gastric cancer by array-based comparative genomic hybridization PLoS One 2011 6 7 e22313 10.1371/journal.pone.0022313 21811585 PMC3141024

[B65] Letessier A Sircoulomb F Ginestier C Cervera N Monville F Gelsi-Boyer V Esterni B Geneix J Finetti P Zemmour C Frequency, prognostic impact, and subtype association of 8p12, 8q24, 11q13, 12p13, 17q12, and 20q13 amplifications in breast cancers BMC Cancer 2006 6 245 10.1186/1471-2407-6-245 17040570 PMC1626089

[B66] Kwek SS Roy R Zhou H Climent J Martinez-Climent JA Fridlyand J Albertson DG Co-amplified genes at 8p12 and 11q13 in breast tumors cooperate with two major pathways in oncogenesis Oncogene 2009 28 17 1892 1903 10.1038/onc.2009.34 19330026 PMC2722962

[B67] Dancau AM Wuth L Waschow M Holst F Krohn A Choschzick M Terracciano L Politis S Kurtz S Lebeau A PPFIA1 and CCND1 are frequently coamplified in breast cancer Genes Chromosomes Cancer 2010 49 1 1 8 10.1002/gcc.20713 19787783

[B68] Tan DS Lambros MB Rayter S Natrajan R Vatcheva R Gao Q Marchio C Geyer FC Savage K Parry S PPM1D is a potential therapeutic target in ovarian clear cell carcinomas Clin Cancer Res 2009 15 7 2269 2280 10.1158/1078-0432.CCR-08-2403 19293255

[B69] Das K Lau W Sivaswaren C Ph T Fook-Chong S Sl T Cheng C Chromosomal changes in prostate cancer: a fluorescence in situ hybridization study Clin Genet 2005 68 1 40 47 10.1111/j.1399-0004.2005.00452.x 15952985

[B70] Zaharieva BM Simon R Diener PA Ackermann D Maurer R Alund G Knonagel H Rist M Wilber K Hering F High-throughput tissue microarray analysis of 11q13 gene amplification (CCND1, FGF3, FGF4, EMS1) in urinary bladder cancer J Pathol 2003 201 4 603 608 10.1002/path.1481 14648664

[B71] Jarmuz M Grenman R Golusinski W Szyfter K Aberrations of 11q13 in laryngeal squamous cell lines and their prognostic significance Cancer Genet Cytogenet 2005 160 1 82 88 10.1016/j.cancergencyto.2004.12.006 15949577

[B72] Hui AB Or YY Takano H Tsang RK To KF Guan XY Sham JS Hung KW Lam CN van Hasselt CA Array-based comparative genomic hybridization analysis identified cyclin D1 as a target oncogene at 11q13.3 in nasopharyngeal carcinoma Cancer Res 2005 65 18 8125 8133 10.1158/0008-5472.CAN-05-0648 16166286

[B73] Wen JM Huang JF Hu L Wang WS Zhang M Sham JS Xu JM Zeng WF Xie D Liang LJ Establishment and characterization of human metastatic hepatocellular carcinoma cell line Cancer Genet Cytogenet 2002 135 1 91 95 10.1016/S0165-4608(01)00636-7 12072206

[B74] Jin C Jin Y Gisselsson D Wennerberg J Wah TS Stromback B Kwong YL Mertens F Molecular cytogenetic characterization of the 11q13 amplicon in head and neck squamous cell carcinoma Cytogenet Genome Res 2006 115 2 99 106 10.1159/000095228 17065789

[B75] Gibcus JH Kok K Menkema L Hermsen MA Mastik M Kluin PM van der Wal JE Schuuring E High-resolution mapping identifies a commonly amplified 11q13.3 region containing multiple genes flanked by segmental duplications Hum Genet 2007 121 2 187 201 10.1007/s00439-006-0299-6 17171571

[B76] Luo ML Shen XM Zhang Y Wei F Xu X Cai Y Zhang X Sun YT Zhan QM Wu M Amplification and overexpression of CTTN (EMS1) contribute to the metastasis of esophageal squamous cell carcinoma by promoting cell migration and anoikis resistance Cancer Res 2006 66 24 11690 11699 10.1158/0008-5472.CAN-06-1484 17178864

[B77] Jin Y Jin C Law S Chu KM Zhang H Strombeck B Yuen AP Kwong YL Cytogenetic and fluorescence in situ hybridization characterization of clonal chromosomal aberrations and CCND1 amplification in esophageal carcinomas Cancer Genet Cytogenet 2004 148 1 21 28 10.1016/S0165-4608(03)00213-9 14697637

[B78] Ishizuka T Tanabe C Sakamoto H Aoyagi K Maekawa M Matsukura N Tokunaga A Tajiri T Yoshida T Terada M Gene amplification profiling of esophageal squamous cell carcinomas by DNA array CGH Biochem Biophys Res Commun 2002 296 1 152 155 10.1016/S0006-291X(02)00836-7 12147242

[B79] Komatsu Y Hibi K Kodera Y Akiyama S Ito K Nakao A TAOS1, a novel marker for advanced esophageal squamous cell carcinoma Anticancer Res 2006 26 3A 2029 2032 16827140

[B80] Kohlhammer H Schwaenen C Wessendorf S Holzmann K Kestler HA Kienle D Barth TF Moller P Ott G Kalla J Genomic DNA-chip hybridization in t(11;14)-positive mantle cell lymphomas shows a high frequency of aberrations and allows a refined characterization of consensus regions Blood 2004 104 3 795 801 10.1182/blood-2003-12-4175 15090459

[B81] Ronchetti D Finelli P Richelda R Baldini L Rocchi M Viggiano L Cuneo A Bogni S Fabris S Lombardi L Molecular analysis of 11q13 breakpoints in multiple myeloma Blood 1999 93 4 1330 1337 9949176

[B82] Fenton JA Pratt G Rothwell DG Rawstron AC Morgan GJ Translocation t(11;14) in multiple myeloma: Analysis of translocation breakpoints on der(11) and der(14) chromosomes suggests complex molecular mechanisms of recombination Genes Chromosomes Cancer 2004 39 2 151 155 10.1002/gcc.10304 14695995

[B83] Tarsitano M Palmieri S Ferrara F Riccardi C Cavaliere ML Vicari L Detection of the t(11;14)(q13;q32) without CCND1/IGH fusion in a case of acute myeloid leukemia Cancer Genet Cytogenet 2009 195 2 164 167 10.1016/j.cancergencyto.2009.08.013 19963117

[B84] Medeiros LJ Carr J Overview of the role of molecular methods in the diagnosis of malignant lymphomas Arch Pathol Lab Med 1999 123 12 1189 1207 10583924 10.5858/1999-123-1189-OOTROM

[B85] Janssen JW Vaandrager JW Heuser T Jauch A Kluin PM Geelen E Bergsagel PL Kuehl WM Drexler HG Otsuki T Concurrent activation of a novel putative transforming gene, myeov, and cyclin D1 in a subset of multiple myeloma cell lines with t(11;14)(q13;q32) Blood 2000 95 8 2691 2698 10753852

[B86] Wlodarska I Meeus P Stul M Thienpont L Wouters E Marcelis L Demuynck H Rummens JL Madoe V Hagemeijer A Variant t(2;11)(p11;q13) associated with the IgK-CCND1 rearrangement is a recurrent translocation in leukemic small-cell B-non-Hodgkin lymphoma Leukemia 2004 18 10 1705 1710 10.1038/sj.leu.2403459 15306823

[B87] de Oliveira FM Tone LG Simoes BP Falcao RP Brassesco MS Sakamoto-Hojo ET dos Santos GA Marinato AF Jacomo RH Rego EM Acute myeloid leukemia (AML-M2) with t(5;11)(q35;q13) and normal expression of cyclin D1 Cancer Genet Cytogenet 2007 172 2 154 157 10.1016/j.cancergencyto.2006.09.004 17213025

[B88] Jhang JS Narayan G Murty VV Mansukhani MM Renal oncocytomas with 11q13 rearrangements: cytogenetic, molecular, and immunohistochemical analysis of cyclin D1 Cancer Genet Cytogenet 2004 149 2 114 119 10.1016/j.cancergencyto.2003.07.001 15036886

[B89] Wells RA Hummel JL De Koven A Zipursky A Kirby M Dube I Kamel-Reid S A new variant translocation in acute promyelocytic leukaemia: molecular characterization and clinical correlation Leukemia 1996 10 4 735 740 8618456

[B90] Wells RA Catzavelos C Kamel-Reid S Fusion of retinoic acid receptor alpha to NuMA, the nuclear mitotic apparatus protein, by a variant translocation in acute promyelocytic leukaemia Nat Genet 1997 17 1 109 113 10.1038/ng0997-109 9288109

[B91] Sukhai MA Wu X Xuan Y Zhang T Reis PP Dube K Rego EM Bhaumik M Bailey DJ Wells RA Myeloid leukemia with promyelocytic features in transgenic mice expressing hCG-NuMA-RARalpha Oncogene 2004 23 3 665 678 10.1038/sj.onc.1207073 14737102

[B92] Imagama S Abe A Suzuki M Hayakawa F Katsumi A Emi N Kiyoi H Naoe T LRP16 is fused to RUNX1 in monocytic leukemia cell line with t(11;21)(q13;q22) Eur J Haematol 2007 79 1 25 31 10.1111/j.1600-0609.2007.00858.x 17532767

[B93] Sarova I Brezinova J Zemanova Z Gancarcikova M Vydra J Cermak J Michalova K A novel gene LRP5 on 11q13.2 is rearranged in two patients with acute myeloid leukemia Leuk Res 2011 35 11 200 20 10.1016/j.leukres.2011.07.022 21821287

[B94] Jesudasan RA Rahman RA Chandrashekharappa S Evans GA Srivatsan ES Deletion and translocation of chromosome 11q13 sequences in cervical carcinoma cell lines Am J Hum Genet 1995 56 3 705 715 7887426 PMC1801173

[B95] Rao PH Harris CP Yan Lu X Li XN Mok SC Lau CC Multicolor spectral karyotyping of serous ovarian adenocarcinoma Genes Chromosomes Cancer 2002 33 2 123 132 10.1002/gcc.1221 11793438

[B96] Wong N Lai P Pang E Leung TW Lau JW Johnson PJ A comprehensive karyotypic study on human hepatocellular carcinoma by spectral karyotyping Hepatology 2000 32 5 1060 1068 10.1053/jhep.2000.19349 11050057

[B97] Yamashita Y Nishida K Okuda T Nomura K Matsumoto Y Mitsufuji S Horiike S Hata H Sakakura C Hagiwara A Recurrent chromosomal rearrangements at bands 8q24 and 11q13 in gastric cancer as detected by multicolor spectral karyotyping World J Gastroenterol 2005 11 33 5129 5135 16127741 10.3748/wjg.v11.i33.5129 PMC4320384

[B98] Shuster MI Han L Le Beau MM Davis E Sawicki M Lese CM Park NH Colicelli J Gollin SM A consistent pattern of RIN1 rearrangements in oral squamous cell carcinoma cell lines supports a breakage-fusion-bridge cycle model for 11q13 amplification Genes Chromosomes Cancer 2000 28 2 153 163 10.1002/(SICI)1098-2264(200006)28:2<153::AID-GCC4>3.0.CO;2-9 10825000

[B99] Martin CL Reshmi SC Ried T Gottberg W Wilson JW Reddy JK Khanna P Johnson JT Myers EN Gollin SM Chromosomal imbalances in oral squamous cell carcinoma: examination of 31 cell lines and review of the literature Oral Oncol 2008 44 4 369 382 10.1016/j.oraloncology.2007.05.003 17681875 PMC2362065

[B100] Mishmar D Rahat A Scherer SW Nyakatura G Hinzmann B Kohwi Y Mandel-Gutfroind Y Lee JR Drescher B Sas DE Molecular characterization of a common fragile site (FRA7H) on human chromosome 7 by the cloning of a simian virus 40 integration site Proc Natl Acad Sci U S A 1998 95 14 8141 8146 10.1073/pnas.95.14.8141 9653154 PMC20943

[B101] Albertson DG Gene amplification in cancer Trends Genet 2006 22 8 447 455 10.1016/j.tig.2006.06.007 16787682

[B102] Reshmi SC Roychoudhury S Yu Z Feingold E Potter D Saunders WS Gollin SM Inverted duplication pattern in anaphase bridges confirms the breakage-fusion-bridge (BFB) cycle model for 11q13 amplification Cytogenet Genome Res 2007 116 1–2 46 52 17268177 10.1159/000097425

[B103] Gajduskova P Snijders AM Kwek S Roydasgupta R Fridlyand J Tokuyasu T Pinkel D Albertson DG Genome position and gene amplification Genome Biol 2007 8 6 R120 10.1186/gb-2007-8-6-r120 17584934 PMC2394771

[B104] Coquelle A Pipiras E Toledo F Buttin G Debatisse M Expression of fragile sites triggers intrachromosomal mammalian gene amplification and sets boundaries to early amplicons Cell 1997 89 2 215 225 10.1016/S0092-8674(00)80201-9 9108477

[B105] Huang X Gollin SM Raja S Godfrey TE High-resolution mapping of the 11q13 amplicon and identification of a gene, TAOS1, that is amplified and overexpressed in oral cancer cells Proc Natl Acad Sci U S A 2002 99 17 11369 11374 10.1073/pnas.172285799 12172009 PMC123263

[B106] Tada K Oka M Tangoku A Hayashi H Oga A Sasaki K Gains of 8q23-qter and 20q and loss of 11q22-qter in esophageal squamous cell carcinoma associated with lymph node metastasis Cancer 2000 88 2 268 273 10.1002/(SICI)1097-0142(20000115)88:2<268::AID-CNCR4>3.0.CO;2-B 10640956

[B107] Takeshita H Ichikawa D Komatsu S Tsujiura M Kosuga T Deguchi K Konishi H Morimura R Shiozaki A Fujiwara H Prediction of CCND1 amplification using plasma DNA as a prognostic marker in oesophageal squamous cell carcinoma Br J Cancer 2010 102 9 1378 1383 10.1038/sj.bjc.6605657 20389301 PMC2865765

[B108] Muller D Millon R Velten M Bronner G Jung G Engelmann A Flesch H Eber M Methlin G Abecassis J Amplification of 11q13 DNA markers in head and neck squamous cell carcinomas: correlation with clinical outcome Eur J Cancer 1997 33 13 2203 2210 10.1016/S0959-8049(97)00198-6 9470807

[B109] Miyamoto R Uzawa N Nagaoka S Nakakuki K Hirata Y Amagasa T Potential marker of oral squamous cell carcinoma aggressiveness detected by fluorescence in situ hybridization in fine-needle aspiration biopsies Cancer 2002 95 10 2152 2159 10.1002/cncr.10929 12412169

[B110] Sugahara K Michikawa Y Ishikawa K Shoji Y Iwakawa M Shibahara T Imai T Combination effects of distinct cores in 11q13 amplification region on cervical lymph node metastasis of oral squamous cell carcinoma Int J Oncol 2011 39 4 761 769 21701773 10.3892/ijo.2011.1094

[B111] Huang X Godfrey TE Gooding WE McCarty KS Jr Gollin SM Comprehensive genome and transcriptome analysis of the 11q13 amplicon in human oral cancer and synteny to the 7F5 amplicon in murine oral carcinoma Genes Chromosomes Cancer 2006 45 11 1058 1069 10.1002/gcc.20371 16906560

[B112] Fortin A Guerry M Guerry R Talbot M Parise O Schwaab G Bosq J Bourhis J Salvatori P Janot F Chromosome 11q13 gene amplifications in oral and oropharyngeal carcinomas: no correlation with subclinical lymph node invasion and disease recurrence Clin Cancer Res 1997 3 9 1609 1614 9815850

[B113] Xia J Chen Q Li B Zeng X Amplifications of TAOS1 and EMS1 genes in oral carcinogenesis: association with clinicopathological features Oral Oncol 2007 43 5 508 514 10.1016/j.oraloncology.2006.05.008 17005439

