# Supplementary material for: Characterization of genetic rearrangements in esophageal squamous carcinoma cell lines by a combination of M-FISH and array-CGH: further confirmation of some split genomic regions in primary tumors
Source: BMC Cancer. 2012 Aug 24;12:367. doi: 10.1186/1471-2407-12-367 (PMC3561653; doi:10.1186/1471-2407-12-367)
Supplement: Additional file 1 — Table S1. Genomic copy number alterations in ESCC cell lines detected by array-CGH. [file 1471-2407-12-367-S1.doc]

**Table S1. Genomic copy number alterations in ESCC cell lines detected by array-CGH**

| **Cell line** | | **Chromosome regions** |
| --- | --- | --- |
| KYSE30 | Gain | 1pter-p36.13, 1p35.2-q23.2, 1q23.2-qter, 2q31.2-q33.1, 3q26.33-qter, 4q13.3-q21.1, 5p14.1-p12, 5q15-qter, 6p22.3-p11, 8q11-qter, 9q22.33-qter, 10q26.3-qter, 11p12-q13.3, 13q21.32-q31.3, 16pter-p11, 17q23.3-qter, 18pter-18q11, 19q12-qter, 20pter-q11.23, 21q11-q21.1 |
|  | Loss | ,2q33.1-q35, 3pter-p11, 4q21.1-q32.1, 6pter-p24.1, 6q11-q13, 6q21, 7p15.1-p14.3, 7q22.2-qter, 9pter-q21.2, 11pter-p12, 11p12, 11q13.4-qter, 14q11-q12, 15q11-q21.1, 18q11-qter, 22q12.3-qter, Xpter-p21.3, Xp21.3-q11, Y |
|  | Amp | 6p24.1-p22.3, 7p12.1-p11, 9q22.1-q22.33, 11q13.3-q13.4, 17p13.1-p11 |
|  | Del | 2p16.2, 4q12, 5q12.2, 5q15, 10p11.21, 21q22.12, Xp21.3 |
| KYSE150 | Gain | 1p22.2-q31.1, 1q44-qter, 2pter-p25.1, 2q35-qter, 3pter-p14.2, 3q11-qter, 7pter-q22.1, 9q11-qter, 11p14.1-qter, 12pter-q14.1, 12q14.1-qter, 16p, 16q, 17p, 17q, 19p, 19q, 20q11-qter, 22 |
|  | Loss | 1q31.1-q32.3, 1q41-q42.2, 1q43-q44, 2p25.1-p24.1, 2q33.2-q35, 3p14.2-p11, 4pter-p14, 4p13-qter, 6pter-p22.2, 6p21.33-p12.2, 6p12.1-qter, 8pter-p22, 9pter-p24.1, 9p23-p11, 10p12.33-qter, 14q21.3-q23.2, 18q11-q21.1, 18q21.1-qter, Xpter-q26.3, Xq27.1-qter |
|  | Amp | 1q32.3-q41, 1q42.2-q43, 4p14-p13, 5p13.1, 6p22.2-p21.33, 6p12.2-p12.1, 9p24.1-p23, 15q11 |
|  | Del | Xq26.3-q27.1 |
| KYSE180 | Gain | 1p, 1q, 2pter-p11, 3q12.2-q12.3, 3q28-qter, 5p, 5q, 6pter-p11, 7pter-q21.3, 8q21.2-q24.22, 9q22.2-qter, 10q25.1-qter, 11pter-p13, 11p13-q13.1, 11q13.3-qter, 12p, 12q, 13q21.32-qter, 14, 15q11-q25.2, 16p, 16q, 17p, 17q, 18pter-q11, 19p, 19q, 20q11-qter, 22, Xq11-q23 |
|  | Loss | 15q25.2, 20p11.23-p11.21, Y |
|  | Amp | 3q13.31-q28, 7q21.3-q22.2, 11q13.1-q13.3 |
|  | Del | 4p15.32-p14, 6q16.3, 9p21.3 |
| KYSE450 | Gain | 1q21.1-q31.1, 2p24.2-q13, 3q12.1-qter, 5pter-p11, 5q32-qter, 7pter-p11, 7q21.13-q22.3, 9q31.1-qter, 11pter-q22.3, 11q23.3, 12pter-q14.4, 15q25.3-qter, 16pter-p11, 16p11-qter, 17q11-qter, 18pter-q12.2, 19p, 19q, 20pter-p12.3, 20q13.31-qter |
|  | Loss | 4q22.1-qter, 8pter-p22, 9q11-q21.13, 11q23.1-q23.3, 11q23.3-qter, 18q12.2-qter, Xq28, Y |
|  | Amp | 5q32, 7p11, 8q24.13-q24.21, 8q24.21, 22q12.2-q12.3, Xq13.1-q13.2 |
|  | Del | 3p14.2, 9p21.3 |
| KYSE510 | Gain | 1q21.2-q23.3, 3q28-qter, 4q34.3-qter, 5pter-q11, 7pter-p12.3, 8q24.12-q24.21, 11p13-p11, 14q22.1-q31.1, 15q25.3-qter, 18q11, 19q12-q13.11, 19q13.11-q13.12, 19q13.12-q13.42, 20q11-qter |
|  | Loss | 1p21.1-q21.2, 2q11-q31.1, 3p14.1-p11, 4pter-p14, 4p13-p11, 4q21.3-q22.1, 6p22.1, 7p12.3-q11.21, 7q21.2-qter, 8q12.1-q23.3, 8q24.21-qter, 9pter-p21.3, 9p21.3-p13.1, 10pter-p11, 11q14.3-q21, 11q22.3-qter, 13q11-qter, 18p11.31-q11, 18q11-q12.1, 18q12.2-qter, 19q13.43-qter, 20pter-p11, 21, X |
|  | Amp | 8q23.3-q24.12, 8q24.21, 11q13.2-q13.4, 11q22.1-q22.3, 18pter-p11.31, 18p11.31, 18q12.1-q12.2, 19q13.11 |
|  | Del | 9p21.3, 19q13.42-q13.43 |
| YES2 | Gain | 2p24.1-q14.3, 3q13.31-q25.2, 3q26.1-q26.2, 3q29-qter, 5pter-p11, 5q21.1-q22.3, 5q23.3-qter, 6p21.1-p12.3, 6p12.3-q16.1, 7pter-q11.21, 7q11.21-q11.22, 7q11.22-q31.1, 7q35-qter, 8q11-q23.3, 9q21.32-qter, 10pter-p11, 11q11-qter, 12pter-q21.1, 13q32.1-qter, 17q11-q25.1, 17q25.3-qter, 18pter-q21.1, 19p, 19q, 20q13.12-qter, Xq21.1 |
|  | Loss | 12q21.1-q21.31, 12q23.1-q24.11, 14q12-q22.1, 18q21.1-qter, Xpter-p11 |
|  | Amp | 3q11-q13.31, 3q26.2-q29, 8q23.3-q24.21, 10q26.3-qter, 11p13-p11, 16p11, 17q25.1-q25.3, 21q11-q21.1 |
|  | Del | 9p21.3, Xq21.1-q23 |

Amp: amplification; Del: deletion
