# Supplementary material for: Characterization of genetic rearrangements in esophageal squamous carcinoma cell lines by a combination of M-FISH and array-CGH: further confirmation of some split genomic regions in primary tumors
Source: BMC Cancer. 2012 Aug 24;12:367. doi: 10.1186/1471-2407-12-367 (PMC3561653; doi:10.1186/1471-2407-12-367)
Supplement: Additional file 2 — Table S2. Regions of gain and the frequencies in the ESCC cell lines. [file 1471-2407-12-367-S2.doc]

**Table S2. Regions of gain and the frequencies in the ESCC cell lines** ^*^

| **Region of gain** | **Frequency** |
| --- | --- |
| 3q26.33-qter | 83% (19/23) |
| 5p14.1-p11 | 87% (20/23) |
| 7pter-p12.3 | 70% (16/23) |
| 8q24.13-qter | 61% (14/23) |
| 9q31.1-qter | 83% (19/23) |
| 11p13-p11 | 61% (14/23) |
| 17q23.3-qter | 78% (18/23) |
| 18pter-p11 | 91% (21/23) |
| 20q13.32-qter | 87% (20/23) |

^*^ Six ESCC cell lines we analyzed (KYSE30, KYSE150, KYSE180, KYSE450, KYSE510 and YES2) and the other seventeen cell lines (KYSE70, KYSE140, KYSE270, KYSE410, KYSE520, Colo-68N, EC-GI-10, HCE-4, TE-1, TE-5, TE-6, TE-8, TE-9, TE-10, TE-11, TE-12 and TE-15).
