# Supplementary material for: Characterization of genetic rearrangements in esophageal squamous carcinoma cell lines by a combination of M-FISH and array-CGH: further confirmation of some split genomic regions in primary tumors
Source: BMC Cancer. 2012 Aug 24;12:367. doi: 10.1186/1471-2407-12-367 (PMC3561653; doi:10.1186/1471-2407-12-367)
Supplement: Additional file 3 — Table S3. Unbalanced breakpoints analyzed according to the copy number alterations. [file 1471-2407-12-367-S3.doc]

**Table S3. Unbalanced breakpoints analyzed according to the copy number alterations**

| **KYSE30** | | **KYSE150** | | **KYSE180** | | **KYSE450** | | **KYSE510** | | **YES2** | |
| --- | --- | --- | --- | --- | --- | --- | --- | --- | --- | --- | --- |
| **Region** | **CN^1^** | **Region** | **CN** | **Region** | **CN** | **Region** | **CN** | **Region** | **CN** | **Region** | **CN** |
| 1p36.3 | G/N | 1p22.2 | N/G | 3q12.2 | N/G | 1q31.1 | G/N | 1p21.1 | N/L | 2p24.1 | N/G |
| 1p35.2 | N/G | 1q31.1 | G/L | 3q12.3 | G/N | 2p24.2 | N/G | 1q21.2 | L/G | 2q14.3 | G/N |
| 1q23.2 | G/G | 1q32.3 | L/A | 3q13.31 | N/A | 2q13 | G/N | 1q23.3 | G/N | 3q13.31 | A/G |
| 2p16.2 | N/D | 1q41 | A/L | 3q28 | A/G | 3p14.2 | N/D | 3p14.1 | N/L | 3q25.2 | G/N |
| 2p16.2 | D/N | 1q42.2 | L/A | 4p15.32 | N/D | 3p14.2 | D/N | 3q28 | N/G | 3q26.1 | N/G |
| 2q31.2 | N/G | 1q43 | A/L | 4p14 | D/N | 3q12.1 | N/G | 4p14 | L/N | 3q26.2 | G/A |
| 2q33.1 | G/L | 1q44 | L/G | 6q16.3 | N/D | 4q22.1 | N/L | 4p13 | N/L | 3q29 | A/G |
| 2q35 | L/N | 2p25.1 | G/L | 6q16.3 | D/N | 5q32 | N/A | 4q21.3 | N/L | 5q21.1 | N/G |
| 3q26.33 | N/G | 2p24.1 | L/N | 7q21.3 | G/A | 5q32 | A/G | 4q22.1 | L/N | 5q22.3 | G/N |
| 4q11 | N/L | 2q33.1 | N/L | 7q22.2 | A/N | 7q21.13 | N/G | 4q34.3 | N/G | 5q23.3 | N/G |
| 4q12 | D/N | 2q35 | L/G | 8q21.2 | N/G | 7q22.3 | G/N | 6p22.1 | N/L | 6p21.1 | N/G |
| 4q13.3 | N/G | 3p14.2 | G/L | 8q24.22 | G/N | 8p22 | L/N | 6p22.1 | L/N | 6p12.3 | G/G |
| 4q21.1 | G/L | 4p14 | L/A | 9p21.3 | N/D | 8q24.13 | N/A | 7p12.3 | G/L | 6q16.1 | G/N |
| 4q32.1 | L//N | 4p13 | A/L | 9p21.3 | D/N | 8q24.21 | A/A | 7q11.21 | L/N | 7q11.21 | G/G |
| 5p14.1 | N/G | 5p13.1 | N/A | 9q22.2 | N/G | 8q24.21 | A/N | 7q21.2 | N/L | 7q11.22 | G/G |
| 5q12.2 | N/D | 5p13.1 | A/N | 10q25.1 | N/G | 9p21.3 | N/D | 8q23.3 | L/A | 7q31.1 | G/N |
| 5q12.2 | D/N | 6p22.2 | L/A | 11p13 | G/G | 9p21.3 | D/N | 8q24.12 | A/G | 7q35 | N/G |
| 5q15 | N/D | 6p21.33 | A/L | 11q13.1 | G/A | 9p13.1 | N/L | 8q24.21 | G/A | 8q23.3 | G/A |
| 5q15 | D/G | 6p12.2 | L/A | 11q13.3 | A/G | 9q21.13 | L/N | 8q24.21 | A/L | 8q24.21 | A/N |
| 6p24.1 | L/A | 6p12.1 | A/L | 13q21.32 | N/G | 9q31.1 | N/G | 9p21.3 | L/D | 9p21.3 | N/D |
| 6p22.3 | A/G | 7q22.1 | G/N | 15q25.2 | G/L | 11q22.3 | G/N | 9p21.3 | D/L | 9p21.3 | D/N |
| 6q13 | L/N | 8p22 | L/N | 15q25.2 | L/N | 11q23.1 | N/L | 11p13 | N/G | 9q21.32 | N/G |
| 6q21 | N/L | 9p24.1 | L/A | 20p11.23 | N/L | 11q23.3 | L/G | 11q13.2 | N/A | 10q26.3 | N/A |
| 6q21 | L/N | 9p23 | A/L | 20p11.21 | L/G | 11q23.3 | G/L | 11q13.4 | A/N | 11p13 | N/A |
| 7p15.1 | N/L | 10p12.33 | N/L | Xq23 | G/N | 12q14.4 | G/N | 11q14.3 | N/L | 12q21.1 | G/L |
| 7p14.3 | L/N | 11p14.1 | N/G |  |  | 15q25.3 | N/G | 11q21 | L/N | 12q21.31 | L/N |
| 7p12.1 | N/A | 12q14.1 | N/G |  |  | 18q12.2 | G/L | 11q22.1 | N/A | 12q23.1 | N/L |
| 7q22.2 | N/L | 14q21.3 | N/L |  |  | 20p12.3 | G/N | 11q22.3 | A/L | 12q24.11 | L/N |
| 7q33 | N/G | 14q23.2 | L/N |  |  | 20q13.31 | N/G | 14q22.1 | N/G | 13q32.1 | N/G |
| 9q21.2 | L/N | 15q11 | A/L |  |  | 22q12.2 | N/A | 14q31.1 | G/N | 14q12 | N/L |
| 9q22.1 | N/A | 18q21.1 | L/N |  |  | 22q12.3 | A/N | 15q25.3 | N/G | 14q22.1 | L/N |
| 9q22.33 | A/G | 18q21.1 | N/L |  |  | Xq13.1 | N/A | 18p11.31 | G/A | 16p11 | N/A |
| 10p11.21 | N/D | Xq26.3 | L/D |  |  | Xq13.2 | A/N | 18p11.31 | A/L | 17q25.1 | G/A |
| 10p11.21 | D/N | Xq27.1 | D/L |  |  | Xq28 | N/L | 18q12.1 | L/A | 17q25.3 | A/G |
| 10q26.3 | N/G |  |  |  |  |  |  | 18q12.2 | A/L | 18q21.1 | G/L |
| 11p12 | L/L |  |  |  |  |  |  | 19q12 | N/A | 20q13.12 | N/G |
| 11p12 | L/G |  |  |  |  |  |  | 19q13.11 | A/L | 21q21.1 | A/N |
| 11q13.3 | G/A |  |  |  |  |  |  | 19q13.11 | L/G | Xq21.1 | N/G |
| 11q13.4 | A/L |  |  |  |  |  |  | 19q13.12 | G/G | Xq21.1 | G/D |
| 13q21.32 | N/G |  |  |  |  |  |  | 19q13.42 | G/D | Xq23 | D/N |
| 13q31.3 | G/N |  |  |  |  |  |  |  |  |  |  |
| 14q12 | L/N |  |  |  |  |  |  |  |  |  |  |
| 15q21.1 | L/N |  |  |  |  |  |  |  |  |  |  |
| 17p13.1 | N/A |  |  |  |  |  |  |  |  |  |  |
| 17q23.2 | N/G |  |  |  |  |  |  |  |  |  |  |
| 19p12 | N/G |  |  |  |  |  |  |  |  |  |  |
| 20q11.23 | G/N |  |  |  |  |  |  |  |  |  |  |
| 21q21.1 | G/N |  |  |  |  |  |  |  |  |  |  |
| 21q22.12 | N/D |  |  |  |  |  |  |  |  |  |  |
| 21q22.12 | D/N |  |  |  |  |  |  |  |  |  |  |
| 22q11.21 | G/N |  |  |  |  |  |  |  |  |  |  |
| 22q12.3 | N/L |  |  |  |  |  |  |  |  |  |  |
| Xp21.3 | L/L |  |  |  |  |  |  |  |  |  |  |
| Xp21.3 | L/L |  |  |  |  |  |  |  |  |  |  |

^1^ Copy number status on the left and right side of the breakpoint regions. CN: copy number, G: gain, L: loss, N: neutral, A: amplification, D: deletion.
