# Supplementary material for: Characterization of genetic rearrangements in esophageal squamous carcinoma cell lines by a combination of M-FISH and array-CGH: further confirmation of some split genomic regions in primary tumors
Source: BMC Cancer. 2012 Aug 24;12:367. doi: 10.1186/1471-2407-12-367 (PMC3561653; doi:10.1186/1471-2407-12-367)
Supplement: Additional file 5 — Table S4. Splitting and amplification of 11q13.3-q13.4 in the primary ESCC tumors by FISH analyses. [file 1471-2407-12-367-S5.doc]

**Table S4. Splitting and amplification of 11q13.3-q13.4 in the primary ESCC tumors by FISH analyses**

| **Case No.** | **Splitting status (11q13.3-q13.4)** | **11q13.3** | | | **11q13.3(1)** | | | **11q13.3(2)** | | | **11q13.4** | | | **11q13.4(1)** | | |
| --- | --- | --- | --- | --- | --- | --- | --- | --- | --- | --- | --- | --- | --- | --- | --- | --- |
|  |  | **Splitting status** | **No. of**  **FISH signals** | | **Splitting status** | **No. of**  **FISH signals** | | **Splitting status** | **No. of**  **FISH signals** | | **Splitting status** | **No. of**  **FISH signals** | | **Splitting status** | **No. of**  **FISH signals** | |
|  |  |  | **16D6** | **2E5** |  | **16D6** | **1D11** |  | **1D11** | **2E5** |  | **2E5** | **15F5** |  | **2E5** | **3C5** |
| 1 | NS | NS | 4 | 4 | NS |  |  | NS |  |  | NS | 4 | 4 | NS |  |  |
| 2 | NS | NS | m | m | NS |  |  | NS |  |  | NS | m | m | NS |  |  |
| 3 | S | S | m-L | m-H | S | m-L | m-H | NS |  |  | S | m | 2 | S | m | 2 |
| 4 | NS | NS | 6 | 6 | NS |  |  | NS |  |  | NS | 6 | 6 | NS |  |  |
| 5 | ND | ND | 6 | 6 | NS |  |  | ND |  |  | NS | 6 | 6 | NS |  |  |
| 6 | NS | NS | 4 | 4 | NS |  |  | NS |  |  | NS | 4 | 4 | NS |  |  |
| 7 | S | NS | 4 | 4 | NS |  |  | NS |  |  | S | 4 | 2 | NS |  |  |
| 8 | NS | NS | 2 | 2 | NS |  |  | NS |  |  | NS | 2 | 2 | NS |  |  |
| 9 | NS | NS | 5 | 5 | NS |  |  | NS |  |  | NS | 5 | 5 | NS |  |  |
| 10 | S | S | 4-5 | m | ND |  |  | ND |  |  | S | m | 4 | S | m | 2-6 |
| 11 | NS | NS | 3 | 3 | NS |  |  | NS |  |  | NS | 3 | 3 | NS |  |  |
| 12 | NS | NS | 3 | 3 | NS |  |  | NS |  |  | NS | 3 | 3 | NS |  |  |
| 13 | S | NS | 2 | 2 | NS |  |  | NS |  |  | S | 4 | 2 | S | 4 | 2 |
| 14 | NS | NS | 3-5 | 3-5 | NS |  |  | NS |  |  | NS | 3-5 | 3-5 | NS |  |  |
| 15 | NS | NS | 3 | 3 | NS |  |  | NS |  |  | NS | 3 | 3 | NS |  |  |
| 16 | NS | NS | 3 | 3 | NS |  |  | NS |  |  | NS | 3 | 3 | NS |  |  |
| 17 | NS | NS | 2 | 2 | NS |  |  | NS |  |  | NS | 2 | 2 | NS |  |  |
| 18 | S | NS | m | m | NS |  |  | NS |  |  | S | m | 2 | ND |  |  |
| 19 | S | S | 2-3 | m | ND |  |  | NS |  |  | S | m | 2-3 | S | m | 2 |
| 20 | NS | NS | 3 | 3 | NS |  |  | NS |  |  | NS | 3 | 3 | NS |  |  |
| 21 | NS | NS | 2 | 2 | NS |  |  | NS |  |  | NS | 2 | 2 | NS |  |  |
| 22 | NS | NS | 2 | 2 | NS |  |  | NS |  |  | NS | 2 | 2 | NS |  |  |
| 23 | NS | NS | 3 | 3 | NS |  |  | NS |  |  | NS | 3 | 3 | NS |  |  |
| 24 | NS | NS | 5 | 5 | NS |  |  | NS |  |  | NS | 5 | 5 | NS |  |  |
| 25 | NS | NS | 2 | 2 | NS |  |  | NS |  |  | NS | 2 | 2 | NS |  |  |
| 26 | S | S | 4 | m | S | 2-5 | m | NS |  |  | S | m | 2 | S | m | 1 |
| 27 | NS | NS | 3 | 3 | NS |  |  | NS |  |  | NS | 3 | 3 | NS |  |  |
| 28 | NS | NS | 5 | 5 | NS |  |  | NS |  |  | NS | 5 | 5 | NS |  |  |
| 29 | NS | NS | 3 | 3 | NS |  |  | NS |  |  | NS | 3 | 3 | NS |  |  |
| 30 | NS | NS | 2 | 2 | NS |  |  | NS |  |  | NS | 2 | 2 | NS |  |  |
| 31 | S | NS | m | m | NS |  |  | NS |  |  | S | m-H | m-L | S | m | 4 |
| 32 | NS | NS | m | m | NS |  |  | NS |  |  | NS | m | m | NS |  |  |
| 33 | S | S | m-L | m-H | S | 5-6 | m | S | m-L | m-H | S | m | 3 | S | m | 3-4 |
| 34 | ND | NS | m | m | NS |  |  | NS |  |  | ND |  |  | ND |  |  |
| 35 | S | NS | 2-5 | 2-5 | NS |  |  | NS |  |  | S | 3 | 2 | NS |  |  |
| 36 | S | S | 4-5 | 2 | NS | 2 | 2 | S | m | 2 | NS | 2 | 2 | NS |  |  |
| 37 | NS | NS | 5 | 5 | NS |  |  | NS |  |  | NS | 5 | 5 | NS |  |  |
| 38 | NS | NS | 3 | 3 | NS |  |  | NS |  |  | NS | 3 | 3 | NS |  |  |
| 39 | NS | NS | 2 | 2 | NS |  |  | NS |  |  | NS | 2 | 2 | NS |  |  |
| 40 | NS | NS | m | m | NS |  |  | NS |  |  | NS | m | m | NS |  |  |
| 41 | S | NS | 3 | 3 | NS |  |  | NS |  |  | S | 3 | 4 | NS |  |  |
| 42 | S | NS | m | m | NS |  |  | NS |  |  | S | m | 1 | S | m | 1-2 |
| 43 | S | S | 2-3 | m | S | 2-4 | m | S | m | 4-7 | NS | 2 | 2 | NS |  |  |
| 44 | NS | NS | m | m | NS |  |  | NS |  |  | NS | m | m | NS |  |  |
| 45 | NS | NS | 4 | 4 | NS |  |  | NS |  |  | NS | 4 | 4 | NS |  |  |
| 46 | S | S | m-L | m-H | NS | m | m | ND |  |  | S | m | 3 | S | m | 3 |
| 47 | NS | NS | 3 | 3 | NS |  |  | NS |  |  | NS | 3 | 3 | NS |  |  |
| 48 | S | NS | m | m | NS |  |  | NS |  |  | S | m | 3 | S | m | 5 |
| 49 | NS | NS | 4 | 4 | NS |  |  | NS |  |  | NS | 4 | 4 | NS |  |  |
| 50 | S | NS | m | m | NS |  |  | NS |  |  | S | m | 2 | S | m | 2 |
| 51 | S | S | 3 | 2 | NS | 3 | 3 | S | 3 | 2 | S | m | 2 | NS | 2 | 2 |
| 52 | S | S | 2 | m | S | 2 | m | NS | m | m | NS | m | m | NS |  |  |
| 53 | S | NS | m | m | NS |  |  | NS |  |  | S | m-H | m-L | NS |  |  |
| 54 | NS | NS | m | m | NS |  |  | NS |  |  | NS | m | m | NS |  |  |
| 55 | S | S | 2-3 | m | S | 2-3 | m | NS | m | m | S | m | 2 | S | m | 2-3 |
| 56 | S | S | 3 | m | S | 3-4 | m | NS | m | m | S | m | 2 | S | m | 2 |
| 57 | S | S | 3 | m | S | 6 | m | NS | m | m | S | m | 2 | S | 4 | 2 |
| 58 | S | NS | m | m | NS |  |  | NS |  |  | S | m | 2-4 | S | m | 2-4 |
| 59 | NS | NS | m | m | NS |  |  | NS |  |  | NS | m | m | NS |  |  |
| 60 | S | S | m-H | m-L | S | 5 | 3 | NS | 5 | 5 | NS |  |  | NS |  |  |
| 61 | S | S | m | 2 | NS | m | m | S | m | 2-3 | NS | 2 | 2 | NS |  |  |
| 62 | NS | NS | 2 | 2 | NS |  |  | NS |  |  | NS | 2 | 2 | NS |  |  |
| 63 | NS | NS | 2-3 | 2-3 | NS |  |  | NS |  |  | NS | 2-3 | 2-3 | NS |  |  |
| 64 | NS | NS | 2-3 | 2-3 | NS |  |  | NS |  |  | NS | 2-3 | 2-3 | NS |  |  |
| 65 | ND | NS | 2-3 | 2-3 | NS |  |  | NS |  |  | ND | 2-3 | 2-3 | NS |  |  |
| 66 | NS | NS | 2-4 | 2-4 | NS |  |  | NS |  |  | NS | 2-4 | 2-4 | NS |  |  |
| 67 | NS | NS | 2 | 2 | NS |  |  | NS |  |  | NS | 2 | 2 | NS |  |  |
| 68 | S | S | 7 | 4 | S | 2-5 | m | S | m | 2 | S | 3 | 4 | S | 3 | 4 |
| 69 | NS | NS | 2 | 2 | NS |  |  | NS |  |  | NS | 2 | 2 | NS |  |  |
| 70 | S | NS | m | m | NS |  |  | NS |  |  | S | m | 2 | NS | m | m |
| 71 | S | NS | m | m | NS |  |  | NS |  |  | S | m | 2 | S | m | 2 |
| 72 | NS | NS | 4 | 4 | NS |  |  | NS |  |  | NS | 4 | 4 | NS |  |  |
| 73 | NS | NS | 5 | 5 | NS |  |  | NS |  |  | NS | 5 | 5 | NS |  |  |
| 74 | S | S | 4 | m | S | 2-7 | m | ND |  |  | S | m | 2 | S | m | 2-3 |
| 75 | S | NS | 4 | 4 | NS |  |  | NS |  |  | S | 6 | 3-4 | NS | 6 |  |
| 76 | NS | NS | 4 | 4 | NS |  |  | ND |  |  | NS | 4 | 4 | NS |  |  |
| 77 | S | NS | m | m | NS |  |  | NS |  |  | S | m | 1 | S | m | 1-2 |
| 78 | NS | NS | 2 | 2 | NS |  |  | NS |  |  | NS | 2 | 2 | NS |  |  |
| 79 | ND | NS | 2 | 2 | NS |  |  | NS |  |  | ND |  |  | ND |  |  |
| 80 | S | NS | 4 | 4 | NS |  |  | NS |  |  | S | 4-5 | 5-6 | NS | 5-6 | 5-6 |
| 81 | S | NS | m | m | NS |  |  | NS |  |  | S | m-H | m-L | S | 8 | 6 |
| 82 | NS | NS | 5 | 5 | NS |  |  | NS |  |  | NS | 5 | 5 | NS |  |  |
| 83 | S | NS | 3-6 | 3-6 | NS |  |  | NS |  |  | S | m-H | m-L | NS | 3-4 | 3-4 |
| 84 | NS | NS | 2 | 2 | NS |  |  | NS |  |  | NS | 2 | 2 | NS |  |  |
| 85 | NS | NS | 2 | 2 | NS |  |  | NS |  |  | NS | 2 | 2 | NS |  |  |
| 86 | S | S | 3-6 | m | S | 2 | 4-7 | NS | m | m | S | m | 2-4 | ND | m | 2-3 |
| 87 | S | S | m | 3 | NS | 3-6 | 3-6 | S | m | 2-3 | NS | 2-3 | 2-3 | NS |  |  |
| 88 | NS | NS | m | m | NS |  |  | NS |  |  | NS | m | m | NS |  |  |
| 89 | S | S | 2 | m | S | 2-3 | m | NS | m | m | S | m | 2-4 | S | m | 2-3 |
| 90 | NS | NS | 4 | 4 | NS |  |  | NS |  |  | NS | 4 | 4 | NS |  |  |
| 91 | S | S | m | 2 | S |  |  | S | m | 2-6 | NS | 2-6 | 2-6 | NS |  |  |
| 92 | S | NS | 3-4 | 3-4 | NS |  |  | NS |  |  | S | 3-4 | 2-3 | NS | 2 | 2 |
| 93 | NS | NS | 2 | 2 | NS |  |  | NS |  |  | NS | 2 | 2 | NS |  |  |
| 94 | NS | NS | 4 | 4 | NS |  |  | NS |  |  | NS | 4 | 4 | NS |  |  |
| 95 | S | NS | m | m | NS |  |  | NS |  |  | S | m | 3 | S | m-H | m-L |
| 96 | NS | NS | 2 | 2 | NS |  |  | NS |  |  | NS | 2 | 2 | NS |  |  |
| 97 | S | S | m-H | m-L | NS | m | m | S | m-H | m-L | S | m | 2-3 | S | m | 2 |
| 98 | S | S | 4 | 2 | S | 4-6 | m | S | m | 2 | NS | 2 | 2 | NS |  |  |
| 99 | NS | NS | 2 | 2 | NS |  |  | NS |  |  | NS | 2 | 2 | NS |  |  |
| 100 | NS | NS | 2-3 | 2-3 | NS |  |  | NS |  |  | NS | 2-3 | 2-3 | NS |  |  |
| 101 | S | S | 5 | m | S | 5 | m | NS | m | m | S | m | 2-4 | S | m | 2 |
| 102 | NS | NS | 5 | 5 | NS |  |  | NS |  |  | NS | 5 | 5 | NS |  |  |
| 103 | S | NS | m | m | NS |  |  | NS |  |  | S | m | 2 | NS | 2 | 2 |
| 104 | S | NS | m | m | NS |  |  | NS |  |  | S | m | 2-3 | S | m | 2 |
| 105 | NS | NS | 2 | 2 | NS |  |  | NS |  |  | NS | 2 | 2 | NS |  |  |
| 106 | S | NS | m | m | NS |  |  | NS |  |  | S | m | 3 | S | m | 2-5 |
| 107 | S | NS | m | m | NS |  |  | NS |  |  | S | m | 2 | NS | 2 | 2 |
| 108 | NS | NS | 3 | 3 | NS |  |  | NS |  |  | NS | 3 | 3 | NS |  |  |
| 109 | NS | NS | 4 | 4 | NS |  |  | NS |  |  | NS | 4 | 4 | NS |  |  |
| 110 | S | S | 3-4 | m | S | 2-5 | m | NS | m | m | S | m | 2 | S | m | 2 |
| 111 | S | S | m-L | m-H | NS | 3 | 3 | ND |  |  | S | m | 2-3 | S | m | 2-3 |
| 112 | S | NS | 4 | 4 | NS |  |  | NS |  |  | S | m | 2 | NS | 5 | 5 |
| 113 | NS | NS | 4 | 4 | NS |  |  | NS |  |  | NS | 4 | 4 | NS |  |  |
| 114 | NS | NS | 5-6 | 5-6 | NS |  |  | NS |  |  | NS | 5-6 | 5-6 | NS |  |  |
| 115 | NS | NS | 4 | 4 | NS |  |  | NS |  |  | NS | 4 | 4 | NS |  |  |
| 116 | S | S | 3-4 | m | S | 2-4 | m | S | m-H | m-L | S | m | 2 | S | m | 2-5 |
| 117 | NS | NS | 4 | 4 | NS |  |  | NS |  |  | NS | 4 | 4 | NS |  |  |
| 118 | NS | NS | 3-4 | 3-4 | NS |  |  | NS |  |  | NS | 3-4 | 3-4 | NS |  |  |
| 119 | NS | NS | 2 | 2 | NS |  |  | NS |  |  | NS | 2 | 2 | NS |  |  |
| 120 | NS | NS | 4 | 4 | NS |  |  | NS |  |  | NS | 4 | 4 | NS |  |  |
| 121 | NS | NS | 3-4 | 3-4 | NS |  |  | NS |  |  | NS | 3-4 | 3-4 | NS |  |  |
| 122 | S | NS | m | m | NS |  |  | NS |  |  | S | m | 3 | NS | 5 | 5 |
| 123 | NS | NS | 4 | 4 | NS |  |  | NS |  |  | NS | 4 | 4 | NS |  |  |
| 124 | NS | NS | 3 | 3 | NS |  |  | NS |  |  | NS | 3 | 3 | NS |  |  |
| 125 | S | S | m-L | m-H | S | 5-6 | m | ND |  |  | S | 5-7 | 2-4 | NS | 3 | 3 |
| 126 | NS | NS | 2-3 | 2-3 | NS |  |  | NS |  |  | NS | 2-3 | 2-3 | NS |  |  |
| 127 | NS | NS | 4 | 4 | NS |  |  | NS |  |  | NS | 4 | 4 | NS |  |  |
| 128 | NS | NS | 4 | 4 | NS |  |  | NS |  |  | NS | 4 | 4 | NS |  |  |
| 129 | NS | NS | 2-m | 2-m | NS |  |  | NS |  |  | NS | 2-m | 2-m | NS |  |  |
| 130 | S | S | m-L | m-H | ND |  |  | NS | m | m | S | m | 2-4 | S | m | 2 |
| 131 | NS | NS | 2-4 | 2-4 | NS |  |  | NS |  |  | NS | 2-4 | 2-4 | NS |  |  |
| 132 | S | S | 2-4 | m | S | m-L | m-H | ND |  |  | S | m | 2 | S | m | 1-2 |
| 133 | S | S | 3-4 | m | S | m-L | m-H | NS | m | m | ND |  |  | ND |  |  |
| 134 | S | S | 4 | m | S | 2-4 | m | NS | m | m | S | m | 1 | S | m | 2 |
| 135 | NS | NS | 6 | 6 | NS |  |  | NS |  |  | NS | 6 | 6 | NS |  |  |
| 136 | NS | NS | m | m | NS |  |  | NS |  |  | NS | m | m | NS |  |  |
| 137 | NS | NS | 2-4 | 2-4 | NS |  |  | NS |  |  | NS | 2-4 | 2-4 | NS |  |  |
| 138 | NS | NS | 3 | 3 | NS |  |  | NS |  |  | NS | 3 | 3 | NS |  |  |

16D6: NONSC16D6; 2E5: NONSC2E5; 1D11: Cancer_1D11; 15F5: NONSC15F5; 3C5: NONSC3C5; S: splitting; NS: non-splitting; ND: not determined; m: multiple FISH signals; m-H (multiple-higher) and m-L (multiple-lower) represent multiple signals of the paired BACs in one FISH experiment. For multiple signals, m-H and m-L are used to distinguish relative higher and lower copy numbers, and breaks may be between two BACs.

11q13.3 (between NONSC16D6 and NONSC2E5) and 11q13.4 (between NONSC2E5 and NONSC15F5) were first detected. If splitting could be observed in the two regions, then small regions, 11q13.3(1), 11q13.3(2) or 11q13.4(1), would be examined and analyzed. Then, splitting of the whole region of 11q13.3-q13.4 would be determined as breakpoints either in 11q13.3 or 11q13.4 could be detected.
