# Supplementary material for: Characterization of genetic rearrangements in esophageal squamous carcinoma cell lines by a combination of M-FISH and array-CGH: further confirmation of some split genomic regions in primary tumors
Source: BMC Cancer. 2012 Aug 24;12:367. doi: 10.1186/1471-2407-12-367 (PMC3561653; doi:10.1186/1471-2407-12-367)
Supplement: Additional file 6 — Table S5. Clinico-pathological parameters of the examined ESCC patients. [file 1471-2407-12-367-S6.doc]

**Table S5. Clinico-pathological parameters of the examined ESCC patients**

| **Case No. ^a^** | **Gender** | **Age** | **pTNM** | **Stage ^b^** | **Grade** |
| --- | --- | --- | --- | --- | --- |
| 1 | M | 70 | T3N1M0 | III | G2 |
| 2 | M | 52 | T3N0M0 | IIa | G1 |
| 3 | M | 39 | T3N1M0 | III | G2/G3 |
| 4 | F | 53 | T2N1M0 | IIb | G2 |
| 5 | M | 82 | T3N1M0 | III | G3 |
| 6 | M | 76 | T3N0M0 | IIa | G1 |
| 7 | M | 53 | T2N1M0 | IIb | G3 |
| 8 | M | 60 | T3N1M0 | III | G1 |
| 9 | M | 58 | T2N1M0 | IIb | G2 |
| 10 | M | 64 | T4N1M0 | III | G1 |
| 11 | M | 58 | T2N0M0 | IIa | G1 |
| 12 | F | 53 | T2N0M0 | IIa | G1/G2 |
| 13 | F | 56 | T2N1M0 | IIb | G1 |
| 14 | M | 56 | T2N0M0 | IIa | G1/G2 |
| 15 | M | 73 | T3N0M0 | IIa | G1 |
| 16 | F | 57 | T3N1M0 | III | G2 |
| 17 | M | 62 | T2N0M0 | IIa | G1 |
| 18 | M | 61 | T3N1M0 | III | G1/G2 |
| 19 | M | 65 | T3N0M0 | IIa | G2 |
| 20 | M | 60 | T4N0M0 | III | G2 |
| 21 | M | 62 | T3N1M0 | III | G3 |
| 22 | M | 44 | T3N1M0 | III | G2 |
| 23 | M | 55 | T3N0M0 | IIa | G2 |
| 24 | F | 45 | T3N0M0 | IIa | G1 |
| 25 | M | 53 | T3N0M0 | IIa | G1/G2 |
| 26 | M | 45 | T3N1M0 | III | G3 |
| 27 | M | 59 | T3N1M0 | III | G2/G3 |
| 28 | M | 55 | T3N0M0 | IIa | G2 |
| 29 | M | 61 | T3N1M0 | III | G1 |
| 30 | F | 58 | T3N0M0 | IIa | G3 |
| 31 | M | 53 | T3N1M0 | III | G2 |
| 32 | M | 60 | T3N1M0 | III | G2 |
| 33 | M | 62 | T3N1M0 | III | G3 |
| 34 | M | 62 | T3N1M0 | III | G2 |
| 35 | M | 55 | T3N0M0 | IIa | G3 |
| 36 | M | 60 | T3N1M0 | III | G2 |
| 37 | M | 68 | T4N1M0 | III | G1/G2 |
| 38 | F | 62 | T3N1M0 | III | G1/G2 |
| 39 | M | 57 | T3N0M0 | IIa | G2/G3 |
| 40 | M | 75 | T3N0M0 | IIa | G2 |
| 41 | M | 71 | T4N1M0 | III | G1 |
| 42 | M | 53 | T3N1M0 | III | G2 |
| 43 | M | 43 | T3N1M0 | III | G2 |
| 44 | M | 62 | T3N1M0 | III | G2 |
| 45 | F | 57 | T3N0M0 | IIa | G2/G3 |
| 46 | M | 58 | T3N1M0 | III | G2 |
| 47 | F | 55 | T3N1M0 | III | G2/G3 |
| 48 | F | 61 | T3N1M0 | III | G1 |
| 49 | M | 52 | T3N1M0 | III | G2 |
| 50 | M | 75 | T2N1M0 | IIb | G2/G3 |
| 51 | F | 63 | T3N1M0 | III | G2 |
| 52 | M | 58 | T3N1M1 | IV | G2 |
| 53 | F | 81 | T3N1M0 | III | G2/G3 |
| 54 | M | 71 | T3N1M0 | III | G2 |
| 55 | M | 48 | T3N1M0 | III | G1 |
| 56 | F | 57 | T3N0M0 | IIa | G1 |
| 57 | M | 54 | T2N0M0 | IIa | G2 |
| 58 | M | 55 | T3N1M0 | III | G2 |
| 59 | F | 66 | T3N0M0 | IIa | G2/G3 |
| 60 | F | 69 | T3N0M0 | IIa | G2 |
| 61 | M | 53 | T3N0M0 | IIa | G1 |
| 62 | M | 50 | T2N0M1 | IV | G2/G3 |
| 63 | M | 66 | T4N1M0 | III | G2 |
| 64 | M | 60 | T3N0M0 | IIa | G1/G2 |
| 65 | M | 57 | T3N1M0 | III | G2 |
| 66 | F | 45 | T3N1M0 | III | G2 |
| 67 | M | 63 | T3N1M0 | III | G1/G2 |
| 68 | M | 69 | T3N1M0 | III | G2 |
| 69 | M | 66 | T3N0M0 | IIa | G2 |
| 70 | M | 55 | T3N1M0 | III | G1/G2 |
| 71 | M | 51 | T2N1M0 | IIb | G1 |
| 72 | M | 65 | T4N0M0 | III | G2 |
| 73 | F | 67 | T2N1M0 | IIb | G1/G2 |
| 74 | M | 70 | T2N0M0 | IIa | G3 |
| 75 | M | 53 | T3N1M0 | III | G2 |
| 76 | M | 52 | T3N1M0 | III | G2 |
| 77 | M | 65 | T3N1M0 | III | G1/G2 |
| 78 | M | 71 | T3N0M0 | IIa | G3 |
| 79 | M | 63 | T3N1M0 | III | G2 |
| 80 | M | 53 | T3N0M0 | IIa | G2 |
| 81 | M | 53 | T4N0M0 | III | G1 |
| 82 | F | 67 | T3N1M0 | III | G2 |
| 83 | M | 56 | T3N1M0 | III | G3 |
| 84 | M | 53 | T3N0M0 | IIa | G1 |
| 85 | M | 50 | T3N0M0 | IIa | G2 |
| 86 | M | 62 | T3N0M0 | IIa | G2/G3 |
| 87 | M | 44 | T3N1M0 | III | G2 |
| 88 | M | 69 | T3N0M0 | IIa | G2 |
| 89 | M | 66 | T3N0M0 | IIa | G2 |
| 90 | F | 66 | T3N0M0 | IIa | G1 |
| 91 | M | 55 | T3N0M0 | IIa | G1/G2 |
| 92 | M | 77 | T4N0M0 | III | G2/G3 |
| 93 | M | 43 | T3N1M0 | III | G3 |
| 94 | F | 59 | T3N0M0 | IIa | G1 |
| 95 | M | 54 | T1N1M0 | IIb | G2 |
| 96 | F | 60 | T3N0M0 | IIa | G1 |
| 97 | M | 56 | T3N1M0 | III | G2/G3 |
| 98 | M | 66 | T3N1M0 | III | G3 |
| 99 | F | 73 | T2N0M0 | IIa | G3 |
| 100 | M | 61 | T3N1M0 | III | G2 |
| 101 | M | 72 | T3N0M0 | IIa | G3 |
| 102 | M | 55 | T3N1M0 | III | G3 |
| 103 | M | 61 | T3N0M0 | IIa | G1 |
| 104 | M | 53 | T3N1M0 | III | G1/G2 |
| 105 | M | 63 | T3N0M0 | IIa | G2 |
| 106 | M | 66 | T4N1M0 | III | G2 |
| 107 | M | 60 | T3N1M0 | III | G2 |
| 108 | M | 55 | T3N0M0 | IIa | G2 |
| 109 | M | 58 | T3N0M0 | IIa | G2 |
| 110 | M | 59 | T3N1M0 | III | G1 |
| 111 | M | 53 | T3N1M0 | III | G2 |
| 112 | M | 70 | T2N0M0 | IIa | G2 |
| 113 | F | 68 | T3N0M0 | IIa | G2 |
| 114 | M | 70 | T3N0M0 | IIa | G1/G2 |
| 115 | M | 52 | T3N1M0 | III | G1 |
| 116 | M | 59 | T3N1M0 | III | G1/G2 |
| 117 | M | 54 | T3N1M0 | III | G1/G2 |
| 118 | M | 66 | T3N0M0 | IIa | G1 |
| 119 | M | 54 | T3N0M0 | IIa | G3 |
| 120 | M | 69 | T3N0M0 | IIa | G3 |
| 121 | M | 42 | T3N0M0 | IIa | G1/G2 |
| 122 | M | 62 | T3N1M0 | III | G3 |
| 123 | M | 67 | T3N1M0 | III | G1 |
| 124 | M | 60 | T3N0M0 | IIa | G1/G2 |
| 125 | F | 53 | T3N1M0 | III | G3 |
| 126 | M | 72 | T3N0M0 | IIa | G1 |
| 127 | F | 70 | T3N1M0 | III | G3 |
| 128 | M | 68 | T4N1M0 | III | G1/G2 |
| 129 | M | 54 | T3N1M0 | III | G1/G2 |
| 130 | F | 50 | T3N0M0 | IIa | G2 |
| 131 | M | 65 | T2N1M0 | IIb | G3 |
| 132 | M | 62 | T3N1M0 | III | G2/G3 |
| 133 | M | 41 | T2N0M0 | IIa | G2 |
| 134 | M | 49 | T1N0M0 | I | G2 |
| 135 | F | 58 | T3N0M0 | IIa | G3 |
| 136 | M | 52 | T3N0M0 | IIa | G2/G3 |
| 137 | M | 52 | T3N0M0 | IIa | G2 |
| 138 | F | 66 | T3N0M0 | IIa | G2 |

M: male, F: female, pTNM: pathological tumor-node-metastasis staging

^a^ Case number is the same as that in Table S4.

^b^ Stage is analyzed according to the sixth edition of American Joint Committee on Cancer (AJCC) cancer staging system for ESCC.
