# Supplementary material for: Characterization of genetic rearrangements in esophageal squamous carcinoma cell lines by a combination of M-FISH and array-CGH: further confirmation of some split genomic regions in primary tumors
Source: BMC Cancer. 2012 Aug 24;12:367. doi: 10.1186/1471-2407-12-367 (PMC3561653; doi:10.1186/1471-2407-12-367)
Supplement: Additional file 7 — Table S6. Relationship between region splittings and clinico-pathological features of ESCC. [file 1471-2407-12-367-S7.doc]

**Table S6. Relationship between region splittings and clinico-pathological features of ESCC**

| **Clinical features** | **11q13.3** | **11q13.4** | **11q13.3(1)** | **11q13.3(2)** | **11q13.4(1)** | **11q13.3(2)**  **-q13.4(1)** | **11q13.3(2)**  **-q13.4** |
| --- | --- | --- | --- | --- | --- | --- | --- |
| Gender | | | | | | | |
| Male | 24.8% (27/109) | 39.6% (42/106) | 17.6% (19/108) | 9.6% (10/104) | 26.7% (28/105) | 33.0% (34/103) | 45.3% (48/106) |
| Female | 17.9% (5/28) | 25.0% (7/28) | 11.1% (3/27) | 3.7% (1/27) | 14.3% (4/28) | 18.5% (5/27) | 25.0% (7/28) |
| *P* value | 0.617 ^a^ | 0.189 ^a^ | 0.565 ^a^ | 0.459 ^a^ | 0.218 ^a^ | 0.165 ^a^ | 0.056 ^a^ |
| Age | | | | | | | |
| < 60 | 27.1% (19/70) | 39.7% (27/68) | 18.8% (13/69) | 9.0% (6/67) | 29.0% (20/69) | 35.8% (24/67) | 44.9% (31/69) |
| ≥ 60 | 19.4% (13/67) | 33.3% (22/66) | 13.6% (9/66) | 7.8% (5/64) | 18.8% (12/64) | 23.8% (15/63) | 36.9% (24/65) |
| *P* value | 0.284 | 0.444 | 0.488 ^a^ | 1.000 ^a^ | 0.168 | 0.135 | 0.347 |
| Tumor size | | | | | | | |
| T1, T2 | 20.0% (4/20) | 47.4% (9/19) | 20.0% (4/20) | 0.0% (0/19) | 36.8% (7/19) | 36.8% (7/19) | 47.4% (9/19) |
| T3, T4 | 23.9% (28/117) | 34.8% (40/115) | 15.7% (18/115) | 9.8% (11/112) | 21.9% (25/114) | 28.8% (32/111) | 40.0% (46/115) |
| *P* value | 1.000 ^a^ | 0.313 ^a^ | 0.742 ^a^ | 0.365 ^a^ | 0.244 ^a^ | 0.589 ^a^ | 0.618 ^a^ |
| Lymph node metastasis | | | | | | | |
| N0 | 20.6% (13/63) | 24.2% (15/62) | 16.4% (10/61) | 3.2% (2/62) | 14.8% (9/61) | 18.3% (11/60) | 27.4% (17/62) |
| N1 | 25.7% (19/74) | 47.2% (34/72) | 16.2% (12/74) | 13.0% (9/69) | 31.9% (23/72) | 40.0% (28/70) | 52.8% (38/72) |
| *P* value | 0.487 | 0.006 | 0.978 | 0.058 ^a^ | 0.025 ^a^ | 0.007 | 0.003 |
| Stage | | | | | | | |
| I, IIa | 22.4% (13/58) | 22.8% (13/57) | 17.9% (10/56) | 3.5% (2/57) | 14.3% (8/56) | 18.2% (10/55) | 26.3% (15/57) |
| IIb, III, IV | 24.1% (19/79) | 46.8% (36/77) | 15.2% (12/79) | 12.2% (9/74) | 31.2% (24/77) | 38.7% (29/75) | 51.9% (40/77) |
| *P* value | 0.823 | 0.004 | 0.679 | 0.112 ^a^ | 0.026 ^a^ | 0.012 | 0.003 |
| Differentiation | | | | | | | |
| G1 | 18.5% (5/27) | 37.0% (10/27) | 14.8% (3/26) | 3.8% (1/26) | 29.6% (8/27) | 33.3% (9/27) | 40.7% (11/27) |
| G2 | 22.7% (17/75) | 32.4% (23/71) | 11.5% (10/73) | 9.6% (7/73) | 22.5% (16/71) | 29.6% (21/71) | 37.5% (27/72) |
| G3 | 28.6% (10/35) | 44.4% (16/36) | 25.0% (9/36) | 9.4% (3/32) | 22.9% (8/35) | 28.1% (9/32) | 48.6% (17/35) |
| *P* value ^b^ | 0.638 | 0.475 | 0.250 | 0.648 | 0.751 | 0.904 | 0.553 |

^a^ Fisher’s test

^b^ Kruskal–Wallis test

The *P* value which is not labeled with “a” or “b” is assessed by χ2 test.
