# Supplementary material for: Characterization of genetic rearrangements in esophageal squamous carcinoma cell lines by a combination of M-FISH and array-CGH: further confirmation of some split genomic regions in primary tumors
Source: BMC Cancer. 2012 Aug 24;12:367. doi: 10.1186/1471-2407-12-367 (PMC3561653; doi:10.1186/1471-2407-12-367)
Supplement: Additional file 8 — Table S8. Relationship between region amplifications and clinico-pathological features of ESCC. [file 1471-2407-12-367-S8.doc]

**Table S7. Relationship between region amplifications and clinico-pathological features of ESCC**

| **Clinical features** | **11q13.3** | | **11q13.4** | |
| --- | --- | --- | --- | --- |
|  | **Frequency** | ***P* value** | **Frequency** | ***P* value** |
| Gender | | | | |
| Male | 85.5% (94/110) | 1.000 ^a^ | 80.4% (86/107) | 0.406 ^a^ |
| Female | 85.7% (24/28) |  | 88.9% (24/27) |  |
| Age | | | | |
| < 60 | 85.7% (60/70) | 1.000 ^a^ | 84.1% (58/69) | 0.540 |
| ≥ 60 | 85.3% (58/68) |  | 80.0% (52/65) |  |
| Tumor size | | | | |
| T1, T2 | 80.0% (16/20) | 0.492 ^a^ | 84.2% (16/19) | 1.000 ^a^ |
| T3, T4 | 86.4% (102/118) |  | 81.7% (94/115) |  |
| Lymph node metastasis | | | | |
| N0 | 79.4% (50/63) | 0.088 ^a^ | 75.4% (46/61) | 0.074 ^a^ |
| N1 | 90.7% (68/75) |  | 87.7% (64/73) |  |
| Stage | | | | |
| I, IIa | 79.3% (46/58) | 0.091 ^a^ | 75.0% (42/56) | 0.070 |
| IIb, III, IV | 90.0% (72/80) |  | 87.2% (68/78) |  |
| Differentiation | | | | |
| G1 | 81.5% (22/27) | 0.159 ^b^ | 77.8% (21/27) | 0.238 ^b^ |
| G2 | 90.7% (68/75) |  | 87.3% (62/71) |  |
| G3 | 77.8% (28/36) |  | 75.0% (27/36) |  |

^a^ Fisher’s test

^b^ Kruskal–Wallis test

The *P* value which is not labeled with “a” or “b” is assessed by χ2 test.
